# Fabrication of a conductive, ionic liquid functionalized, oxygen-generating hydrogel loaded with ADSC-derived exosomes to enhance angiogenesis and cardiac repair after acute myocardial infarction

**DOI:** 10.7150/thno.118652

**Published:** 2026-01-01

**Authors:** Zhaoyan Xu, Jian Chen, Ling Shu, Wanzi Hong, Yuanxi Mo, Jiaqi Wang, Yaoxin Liu, Wing-Tak Wong, Suk-Ying Tsang, Ning Tan, Lei Jiang

**Affiliations:** 1 Department of Cardiology, Guangdong Provincial People's Hospital (Guangdong Academy of Medical Sciences), Southern Medical University, Guangzhou 510080, China.; 2 Department of Cardiology, The First People's Hospital of Foshan, Foshan 528000, China.; 3 SDU-ANU Joint Science College, Shandong University, Weihai 264209, China.; 4 Department of Applied Biology and Chemical Technology, The Hong Kong Polytechnic University, Hong Kong, China.; 5 School of Life Sciences, The Chinese University of Hong Kong, Hong Kong SAR, China.

**Keywords:** injectable hydrogel, exosomes, conductive polymer, oxygen generation, myocardial infarction therapy

## Abstract

Acute myocardial infarction (AMI) remains one of the most severe and life-threatening cardiac diseases, accounting for a substantial proportion of mortality worldwide and necessitating the development of novel treatment approaches for effective myocardial repair. Biocompatible, ionically conductive injectable hydrogel scaffolds have emerged as promising candidates for AMI treatment owing to their prominent electrical conductivity and mechanical compatibility with cardiac tissue.

**Methods:** In the present investigation, a facile ionic-conductive injectable hydrogel was developed by incorporating the conductive polymer PEDOT and boronic acid-functionalized carboxymethyl chitosan (pCMC) with poly-ionic liquid (P-ILs), oxygen-generating calcium peroxide (CaO_2_) particles, and adipose-derived stem cell exosomes (ADSC-exos) to improve myocardial regeneration via enhancing conductivity and oxygen release. The physiological and mechanical characteristics of the injectable hydrogel were examined through morphological evaluation, oxygen-release profiling, electroactivity, and rheological analysis. *In vitro* biocompatibility was assessed using rat cardiac fibroblasts (RCF) and H9C2 cells. For *in vivo* investigation, a rat ischemia-reperfusion model was established, and the developed hydrogels were administered for 28 days to examine cardiac function recovery and myocardial regeneration.

**Results:** The encapsulation of Exo within the hydrogel enhanced its biological performance by promoting antioxidant activity and angiogenesis, while concurrently inhibiting cardiomyocyte (CM) apoptosis and inflammation. Both *in vitro* and *in vivo* findings demonstrated that the Exo-loaded ICon-O_2_-hydrogel significantly enhanced reactive oxygen species (ROS) scavenging, cellular proliferation, migration, angiogenesis responses, resulting in effective myocardial repair, accelerated cardiac functional recovery, reduced fibrosis, and increased neovascularization in the rat model.

**Conclusion:** The combined therapeutic model integrating ADSC-exos with an oxygen-generating ionic-conductive hydrogel demonstrates a promising potential for AMI therapy by reducing fibrosis and scar formation while promoting angiogenesis and myocardial regeneration.

## Introduction

Acute myocardial infarction (AMI) is a severe cardiovascular disease caused by coronary artery occlusion, resulting in local ischemia and hypoxia. The pathological progression of AMI involves cardiomyocyte (CM) necrosis, fibrotic tissue formation within the infarcted area, and ventricular remodeling, which can lead to persistent ischemia, malignant arrhythmia, and eventual heart failure 1,2. The non-conductive fibrotic tissue formed in the infarcted myocardium creates a proarrhythmic and heterogeneous environment primarily due to the electrical decoupling of viable CMs. The loss of electrical continuity between fibrotic and healthy myocardium impairs ventricular contraction, resulting in progressive functional decompensation 3. Recent studies have indicated that restoring cardiac impulse propagation by re-establishing electrical connectivity between CMs and non-fibrotic tissues can prevent ventricular remodeling and synchronize contraction. Although current clinical interventions can delay mortality, they are insufficient to prevent ventricular remodelling. Heart transplantation remains the ultimate option but faces significant limitations due to donor scarcity and high treatment costs 4,5.

Extensive efforts have therefore been made to develop biocompatible and functionally appropriate biomaterials to substitute the damaged extracellular matrix in the infarcted myocardium 6,7. Conductive biomaterials are particularly beneficial, as they promote CM maturation and intercellular communication, thereby supporting synchronized electrical activity. Numerous studies have focused on developing suitable conductive biomaterials with appropriate injectability and mechanical properties for AMI therapy, facilitating cardiac regeneration and functional repair 8-10. Conductive nanomaterials such as graphene, conductive polymers, metallic nanoparticles, and carbon nanotubes have demonstrated enhanced electrical performance; however, concerns regarding their long-term biocompatibility, biodegradability, and metabolic safety after myocardial injection remain 11-14. To address these issues, a conductive biomaterial was developed using a bio-ionic liquid (BIL) with suitable biocompatibility, ionic conductivity, and electrochemical stability. Poly-ionic liquids (P-ILs), a class of polymerized small molecules, possess excellent ionic conductivity, water solubility, low melting points, and high electrochemical stability. P-ILs have been explored for various biological applications, including drug delivery and tissue regeneration. Recent reports have described BIL-grafted hydrogels exhibiting tunable electrical and mechanical properties with good biodegradability and low immunogenicity. Based on this evidence, 1-vinyl-3-butylimidazolium bromide ([VBIM]Br) was selected as a suitable BIL to be incorporated into an oxygen-generating hydrogel, aiming to synergistically improve electrical stimulation and oxygen supply for myocardial repair 15,16.

In recent decades, stem cell-based therapies have been widely investigated for cardiac repair in both preclinical and clinical settings. However, their therapeutic efficacy remains limited due to poor cell retention and survival within the ischemic myocardium, largely attributed to oxidative stress and oxygen deficiency 17-19. Increasing evidence suggests that the beneficial effects of stem cell therapy are closely associated with the paracrine functions of secreted exosomes. Exosomes, naturally secreted nanosized vesicles derived from stem cells, exhibit regenerative and reparative capabilities comparable to their parental cells. Mesenchymal stem cell (MSC)-derived exosomes incorporated into injectable hydrogels have emerged as an alternative, cell-free approach that enhances cardiac regeneration following AMI 20-23.

**Figure 1:**
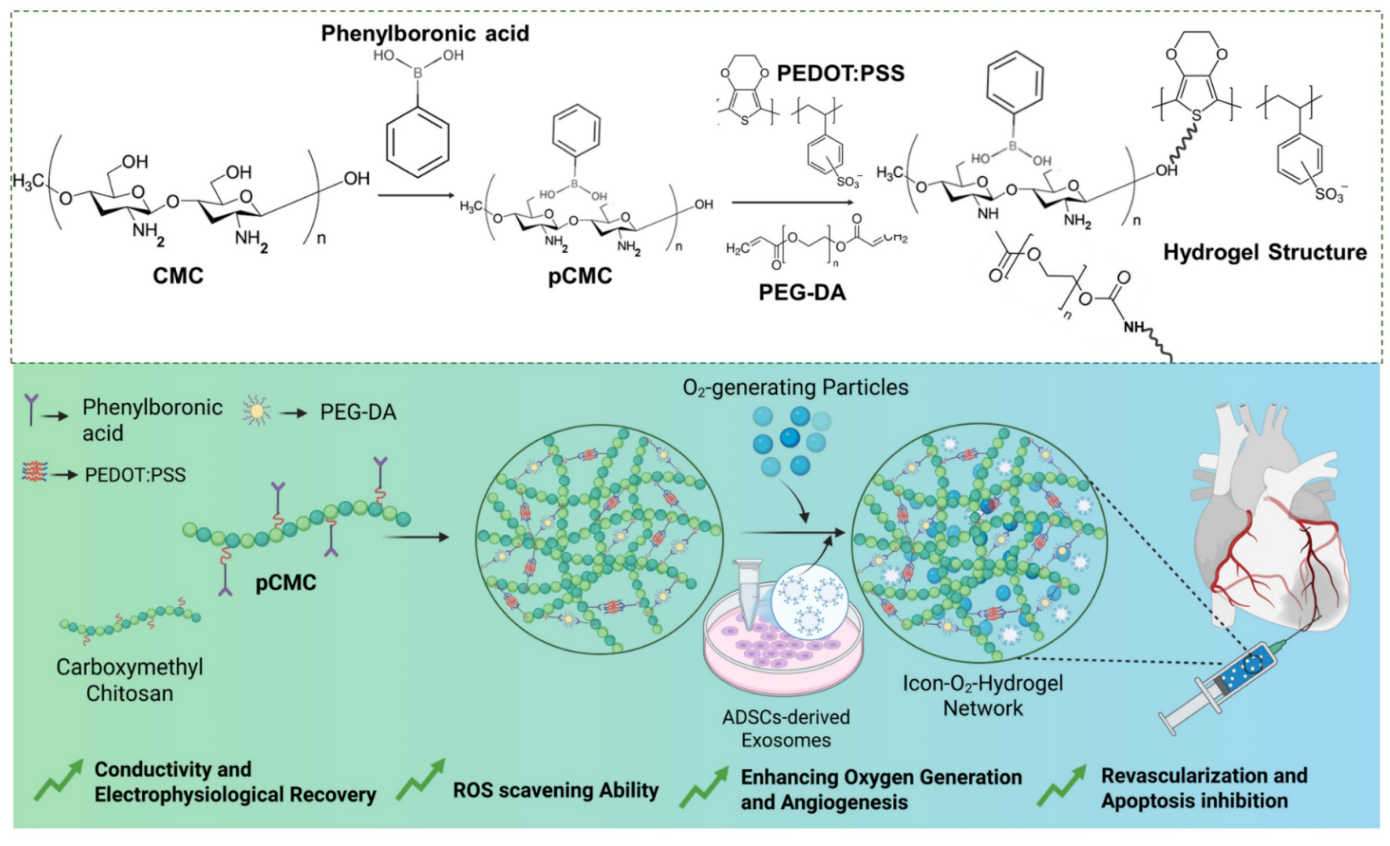
Chemical interactions among the major components of the hydrogel and schematic illustration of the exosome-loaded conductive oxygen-generating injectable hydrogel and its proposed mechanism for cardiac repair following acute myocardial infarction.

Given that the myocardium has one of the highest oxygen demands in the body, requiring approximately 70 mL O_2_/min/100 g of tissue during normal cardiac activity, oxygen generation within cardiac biomaterials is vital for AMI treatment. Adequate oxygen delivery supports tissue repair and mitigates hypoxia-induced necrosis 24. Sustained oxygen release from injectable hydrogels further promotes CM survival, differentiation, and proliferation during tissue recovery 25,26. In the present study, a biopolymeric injectable hydrogel composed of poly(3,4-ethylenedioxythiophene) (PEDOT), carboxymethyl chitosan (CMC), [VBIM]Br, and calcium peroxide (CaO_2_) particles was fabricated (Figure 1). PEDOT, a conjugated polymer, has been recognized for its strong interaction with CM membranes, enhancing proliferation, differentiation, and electrophysiological activity 27, 28. Adipose-derived stem cell exosomes (ADSC-exos) were incorporated into the hydrogel to provide additional therapeutic benefit. These exosomes possess anti-inflammatory, pro-angiogenic, and cytoprotective effects, and contain bioactive molecules such as proteins, lipids, and miRNAs that regulate gene expression to reduce apoptosis and promote tissue repair. In addition to that, ADSC-exos carry immunomodulatory factors (e.g., TGF-β, miR-146a) and pro-angiogenic mediators (e.g., VEGF, miR-126), which help suppress macrophage infiltration, alleviate inflammation, and promote neovascularization, thereby minimizing further myocardial damage. The injectable hydrogel serves as a depot for the sustained release of exosomes at the infarct site. Consequently, the exosome-loaded, oxygen-generating ionic-conductive hydrogel is expected to improve blood perfusion, oxygenation, and nutrient delivery to support myocardial regeneration. The fabricated hydrogel was injected into AMI-induced rat hearts one week post-infarction, and its effects on arrhythmia prevention, ventricular protection, and impulse propagation were evaluated. The therapeutic efficacy was assessed through *in vitro* CM models and *in vivo* rat studies. This study hypothesized that the ionic-conductive hydrogel, through its oxygen-generating and exosome-releasing properties, can attenuate myocardial fibrosis and promote cardiac repair. To the best of current knowledge, this is the first report describing an injectable hydrogel that integrates both electrical conductivity and oxygen-generating capacity using BILs for AMI therapy.

## Materials and Methods

### Chemicals and Reagents

Polymeric components, including 3,4-ethylenedioxythiophene (EDOT, 97 %) and poly(ethylene glycol) diacrylate (PEG, Mw 700), were purchased from Sigma-Aldrich (USA). Ammonium persulfate, 4-formylphenylboronic acid, and 1,1-diphenyl-2-picrylhydrazyl (DPPH) were obtained from Aladdin (PR China). Chitosan (90% degree of deacetylation), extracted from shrimp and crab shells, was purchased from Sigma-Aldrich (USA). Monochloroacetic acid, sodium hydroxide (NaOH), 4-formylphenylboronic acid, isopropanol, and sodium borohydride (NaBH_4_) were procured from Shanghai Macklin Biochemical Co., Ltd. (China). Biological kits and fluorescent staining reagents were obtained from Sigma-Aldrich (USA) and Aladdin Reagent (Shanghai, China).

### Preparation of phenylboronic acid-functionalized carboxymethyl chitosan (PBA-CMC)

CMC was prepared following previously reported methods with minor modifications^29^. The synthesis involved two sequential steps—alkalization and etherification—under heterogenous conditions using a cylindrical glass reactor equipped with a thermostat maintained at 25 ± 2 °C. Purified chitosan powder (5 g) was dissolved in 50% aqueous NaOH (w/v) and 65 mL isopropanol under magnetic stirring for 15 min at room temperature, followed by overnight incubation at -20 °C to complete alkalization. The alkalized chitosan was then reacted with 9 g monochloroacetic acid in isopropanol under constant stirring for 10 h at room temperature. The resulting solid precipitate was filtered, washed with methanol, neutralized using glacial acetic acid, and further washed with 80% ethanol to eliminate residual by-products before air-drying. The purified CMC was dialyzed against deionized (DI) water for five days at room temperature and freeze-dried to obtain the final product. Subsequently, 1 g of CMC was dissolved in 100 mL DI water and reacted 4-formylphenylboronic acid (1 mmol) under stirring for 12 h at room temperature via a Schiff-base reaction. NaBH_4_ was then added dropwise, and the mixture was allowed to react for an additional 3 h at 4 °C. The reaction mixture was dialyzed against DI water using a molecular weight cut-off membrane (Mw = 80 k-100 k) for three days, and the purified phenylboronic acid-functionalized CMC (PBA-CMC) was obtained by vacuum-drying at 50 °C for subsequent use.

### Preparation of ionic-conductive hydrogel

The injectable hydrogel was fabricated through supramolecular interactions between CMC and the PEDOT conductive polymer. In brief, a PBA-CMC reaction solution was prepared by dissolving synthesized CMC powder (4 wt%) in a phosphate-buffered saline (PBS)/DI water mixture (1:1, v/v) under magnetic stirring for 12 h at room temperature. Then, 2% (w/v) of EDOT monomer (0.2 g) and ammonium persulfate (APS, 1.5 eq.) were added to the PBA-CMC solution and stirred vigorously for 12 h to initiate in-situ polymerization. Following this, 0.3 M PIL ([VBIM]Br), 5 wt% PEG-DA (0.5 g), and 1 mg/mL calcium peroxide (CaO_2_) were incorporated into the reaction mixture to promote gelation at room temperature, which occurred within approximately 2-3 min. The resulting oxygen-generating ionic-conductive hydrogel (ICon-O_2_-Hydrogel) was then freeze-dried at -40 °C for further characterization. For comparative analysis, an ionic-conductive hydrogel without the oxygen-generating component (CaO_2_) was also prepared and labelled as the ICon-hydrogel group.

### Characterization of hydrogel

The cross-sectional morphology and exosome distribution within the ICon-O_2_-Hydrogel were examined using field-emission scanning electron microscopy (FE-SEM; Zeiss Supra 40VP, Germany) and transmission electron microscopy (TEM; JEM 1400 PLUS, JEOL, Germany). Mechanical properties of the prepared hydrogel were evaluated through rheological analysis (Discovery DHR-2 Rheometer, TA Instruments, USA) and gelation behavior studies. A lid was utilized during all the rheological measurements to minimize sample evaporation. Oscillatory deformation within the linear viscoelastic regime was confirmed using a frequency of 1 Hz and a strain of 5%. Time-sweep tests were performed at 1 Hz and 37 °C over a duration of 0-10 min, while frequency-sweep measurements were conducted between 0.1 and 20 Hz at a constant stress of 20 Pa and 37 °C. Fatigue resistance was assessed at 5 Hz and 37 °C over 10 h. The biodegradation behavior of the ICon-O_2_-Hydrogel was determined by measuring the mass of freeze-dried scaffolds before and after incubation in PBS (pH 7.4) at 37 °C over predetermined time intervals. The percentage of mass loss was calculated using the following equation:


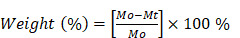

(1)

where 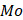
and 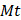
represent the initial and remaining dry weights of the hydrogel, respectively.

### *In vitro* oxygen-releasing profile

The cumulative oxygen-releasing capacity of the ICon-O_2_-Hydrogel was evaluated under hypoxic cell culture conditions as previously reported 30. The percentage of dissolved oxygen was measured at defined time intervals over 24 days using a ruthenium-complex oxygen-sensing probe (Ocean optics, NeoFax system). The probe was placed on culture media containing the hydrogel, both in the presence and absence of cells. Oxygen levels within the incubator were regulated by a hypoxia chamber, ensuring that the oxygen-generating hydrogel served as the primary oxygen source. To enhance oxygen generation, 1 mg mL^-1^ of catalase was added to the reaction medium, as this enzyme decomposes hydrogen peroxide (H_2_O_2_) into oxygen and water, enhancing oxygen yield under hydrolysis. Evaluation under these conditions was essential to optimize oxygen release behavior from the oxygen-generating scaffolds.

### Extraction of exosomes from ADSCs

Exosomes were extracted from ADSCs harvested from 5-week-old male Sprague-Dawley (SD) rats (200±30)g according to previously described protocols 31. Rats were anesthetized by Sodium pentobarbital 50mg/kg intraperitoneal anesthesia and euthanized by cervical dislocation in accordance with institutional ethical guidelines. After disinfection, the abdominal cavity was surgically exposed under aseptic conditions, and ophthalmic forceps were used to isolate the testes together with the surrounding epididymal adipose tissue while avoiding damage to associated blood vessels. The excised adipose tissue was immediately transferred into sterile PBS (pH 7.4), rinsed three times to remove residual blood, and clipped to separate connective tissue.

The collected adipose tissue was incubated with 0.10 % (w/v) type I collagenase (Sigma-Aldrich) in a shaking water bath at 37 °C for 1 h at 120 rpm. The digestion medium was neutralized by adding an equal volume of complete culture medium (DMEM containing 10% FBS). The suspension was centrifuged at 1600 r/min for 10 min, and the cell pellet was in fresh culture medium, filtered through a 70 µm cell sieve, and seeded into culture flasks. ADSCs were cultured in DMEM supplemented with 10% exosome-depleted FBS and 1% penicillin/streptomycin at 37°C in a humidified 5% CO₂incubator. The culture medium was replaced every 48 h, and cells between passages 3 and 6 were used for subsequent experiments. Flow-cytometric analysis confirmed positive expression of CD29 and CD44 and absence of CD34 surface marker. For exosome isolation, ADSCs were cultured in serum-free medium for 48 h, and the collected supernatant was centrifuged at 3500 rpm for 20 min to eliminate cell debris, followed by centrifugation at 10000 g (4 °C, 30 min) to remove larger vesicles. Exosomes were finally obtained by ultracentrifugation at 100 000 g for 80 min and resuspended in PBS for subsequent use.

### Preparation of exosome-loaded hydrogel

To visualize exosomes incorporated into the ICon-O_2_-Hydrogel, isolated exosomes were stained with DiR fluorescent dye. A 5 µM working solution of DiR in serum-free medium (prepared from a DMSO stock) was incubated with ADSC-exos at room temperature for 25 min. The stained exosomes were ultracentrifuged at 100 000 g for 30 min to remove unbound dye and resuspended in PBS. The purified, fluorescently labelled exosomes were mixed into the pre-gel hydrogel solution before gelation and designated as Exo@ICon-O_2_-Hydrogel. Following gelation, exosome distribution within the hydrogel was visualized by confocal microscopy.

### Analysis of hydrogel electroactivity

The electroconductivity of the prepared hydrogel groups was evaluated using three complementary methods as described previously 5. Initially, the electrical conductivity was measured using the Van Der Pauw four-probe technique using hydrogel scaffolds (0.5 cm × 0.5 cm). Subsequently, electrochemical measurements were performed on a conventional three-electrode electrochemical workstation (CHI660D, CH Instruments, USA) to evaluate the electroactivity of the hydrogels. The three-electrode configuration is a commonly adopted method for conductivity assessment of biological hydrogels designed for myocardial infarction therapy. This system provides accurate and reproducible measurements with minimal noise and controlled current-potential response, and is well suited for soft, biocompatible materials. Cyclic voltammetry (CV) and electrochemical impedance spectroscopy (EIS) were employed to characterize the conductive behavior of the hydrogels. In this configuration, the hydrogel was applied to the working electrode, the reference electrode maintained a stable potential, and the counter electrode completed the circuit to permit current flow 9,10. Prior to testing, the fabricated hydrogels were saturated with anhydrous ethanol to reach equilibrium and subsequently dried in an oven. CV analysis was then carried out using hydrogel-coated glassy-carbon working electrode, a platinum sheet as the counter electrode, and a Hg/HgCl_2_ electrode as the reference. The electrolyte solution consisted of 0.1 mol/L PBS (pH 7.4) at room temperature. The potential was scanned from -0.8 V to +0.8 V of 100 mV/s to record the electrochemical response of the hydrogels.

### Study on ROS-scavenging ability

The antioxidant efficiency and ROS-scavenging capability of the prepared hydrogels were evaluated using DPPH and hydroxyl radical assays. For DPPH radical scavenging, a uniform organic solution of DPPH (0.1 mM) was prepared in ethanol. Freeze-dried hydrogel scaffolds were immersed in 2 mL of the prepared DPPH solution in centrifuge tubes and incubated at 25 °C under dark conditions for a predetermined time period. After incubation, the samples were centrifuged, and the absorbance of the collected supernatant was measured at 517 nm using a UV-visible spectrophotometer. The DPPH radical-scavenging efficiency was calculated according to the following equation:


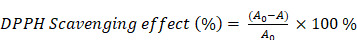

(2)

where *A* and *A_0_* represent the absorbance of the untreated DPPH solution and the DPPH solution treated with hydrogel samples, respectively.

The hydroxyl radical scavenging ability of the hydrogels was further examined based on the Fenton reaction, as described previously 32,33. In brief, FeSO_4_ solution (600 µL; 2 mM) and safranin O solution (500 µL; 350 µg/mL) were mixed and then treated with hydrogel samples (300 µL). The mixture was incubated for 15 min, after which 800 µL H_2_O_2_ solution (6 wt%) was added. The resulting reaction mixture was incubated at 55 °C for 30 min, cooled to room temperature, and the absorbance was recorded at 492 nm using a microplate reader (Synergy HT, Bio Tek, USA). The hydroxyl radical scavenging efficiency was determined using the following equation:


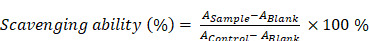

(3)

where *A_sample_*, *A_Blank_*_,_ and *A_Control_* correspond to the absorbance values of the hydrogel-treated sample, the blank and the control groups respectively.

### *In-vitro* assays for cardiac cell suitability

#### *In-vitro* cardiac cell compatibility assay

The cardiac cell viability and compatibility of the prepared conductive hydrogel groups were evaluated by the MTT assay using rat cardiac fibroblasts (RCFs) and H9C2 cardiomyocytes (CMs) as model cell lines. The selected cells were seeded into 96-well plates at a density of 5 × 10^3^ cells per well in optimized culture medium and incubated under standard conditions (37 °C, 5 % CO_2_, humidified atmosphere) for 24 h. After initial attachment, the culture medium was discarded and replaced with 100 µL fresh medium containing the hydrogel extract. Following 24 h and 48 h of incubation, 20 µL of MTT solution (5 mg/mL) was added to each well and incubated for an additional 4 h. The supernatant was then carefully removed and replaced with 150 µL of DMSO to dissolve the formazan crystals. The plates were gently shaken for 30 min, and absorbance was recorded at 570 nm using a Synergy HT microplate reader (Bio Tek, USA). Untreated cells served as the control group. The percentage of cell viability (%) was calculated using the following equation:


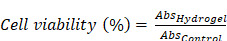
× 100 (4)

where Abs_Hydrogel_ and Abs_Contol_ represent the absorbance values of the hydrogel-treated and control (untreated) groups, respectively.

#### *In vitro* cell proliferation assay

The cell proliferation efficacy of the prepared hydrogel was qualitatively examined using fluorescence staining. Cells were seeded into 12-well plates at a density of 2 × 10^4^ cell per well and treated with hydrogel extracts. The samples were incubated for predetermined periods of 24, 48 and 72 h under standard culture conditions (37 °C, 5 % CO_2_, humidified atmosphere). At each interval, Calcein-AM solution prepared in PBS was added to the treated wells and incubated for 15 min at room temperature. Fluorescence images were captured using an Olympus CKX53 fluorescence microscope (Olympus, Japan).

#### *In vitro* wound healing experiment

The migratory ability of the hydrogel-treated cells was evaluated using a scratch wound healing assay with human umbilical vein endothelial cells (HUVECs). Cells were seeded into 6-well plates and cultured under standard conditions until reaching approximately 95% confluence. A straight and uniform scratch was created in the cell monolayer using a sterile 200 µL pipette tip, followed by rinsing with PBS to remove detached cells. The scratched wells were immediately supplemented with medium containing hydrogel extracts and incubated for 48 h. Cell migration before and after treatment was visualized using acridine orange (AO) staining and imaged with an Olympus CKX53 fluorescence microscope. The migration percentage (%) was quantified using ImageJ software (NIH, USA).

#### *In vitro* tube formation assay

The pro-angiogenic efficiency of the prepared Exo@ICon-O_2_-Hydrogel was examined using the Matrigel^®^ tube formation assay on HUVECs. Briefly, 10 µL of Matrigel^®^ (Invitrogen, USA) was added to each well of an Ibidi^®^ angiogenesis plate (Germany) and incubated at 37 °C for 30 min to allow complete gelation. Subsequently, HUVECs were seeded into each well at a density of 5 × 10^4^ cells per well, and cultured in endothelial growth medium containing the respective hydrogel formulations. After incubation, tube formation was visualized by fluorescence microscopy (Olympus CKX53, Japan) following Calcein-AM staining. Tube formation parameters, including total tube length and number of junctions, were analyzed at different time intervals using the Angiogenesis Analyser plugin in ImageJ software.

#### *In vitro* intracellular antioxidant study

H9C2 CMs were seeded in 24-well plates at a density of 5 × 10^4^ cells per well in high-glucose DMEM and cultured for 12 h to allow proper attachment. The culture medium was then replaced with 500 µL of fresh culture medium containing H_2_O_2_ to induce oxidative stress. Subsequently, the prepared hydrogel formulations were added to each well and co-cultured for an additional 6 h. After treatment, the cells were incubated with DCFH-DA or DHE staining solution for 30 min, followed by DAPI counterstaining for 10 min. Intracellular ROS levels and fluorescence signals were visualized using an Olympus CKX53 fluorescence microscope (Olympus, Japan). In addition, the antioxidant capability of the prepared hydrogels against oxidative stress was further evaluated in H9C2 CMs using the CCK-8 assay under H_2_O_2_-induced conditions.

#### *In vitro* hemolysis assay

The *in vitro* hemolytic potential of the prepared conductive hydrogels was evaluated using a rabbit erythrocyte suspension, following a previously described method 12. Hydrogel extracts were first prepared by immersing samples in sterile saline at 37 °C for 24 h. Then, 2.5 mL of each hydrogel extract was mixed with 2.5 mL of a 2% rabbit erythrocyte suspension and incubated at 37 °C for 3 h. After incubation, the mixtures were centrifuged at 1600 rpm for 15 min, and the absorbance of the supernatant was measured at 545 nm using a spectrophotometer. DI water and sterile saline served as the positive and negative controls, respectively. The hemolytic ratio (%) was calculated using the following equation:


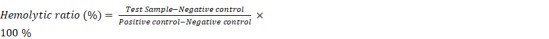

(5)

#### *In vitro* biodegradation of hydrogel

To simulate *in vivo* degradation behaviour, *in vitro* degradation of the hydrogels was evaluated in the presence of collagenase type II. Freeze-dried hydrogel scaffolds (disk-shaped; thickness 2 mm, diameter 10 mm) were weighed (W_0_) before testing and incubated in 10 mL PBS solution (pH 7.4) containing collagenase type II (25 U mL^-1^) and calcium ions (1.5 mM) at 37 °C. The degradation medium was replaced at predetermined intervals to maintain enzyme activity. At each designated time point (1, 3, 5, and 7 weeks), the samples were carefully removed, washed with DI water, and freeze-dried. The dry weight (W_t_) of each sample was recorded, and the degradation ratio was calculated using the following equation:


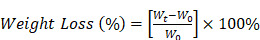

(6)

#### Analysis of cardiac protein expressions in CMs

The expression of cardiac-specific proteins, including connexin 43 (Cx43) and sarcomeric α-actinin (SARC)), was analyzed in CMs cultured on hydrogel-coated substrates suing immunofluorescence staining. Briefly, CMs was seeded on hydrogel-coated culture disks and incubated under standard culture conditions for 3 days. Cells were then fixed with 2% paraformaldehyde and blocked with 5% donkey serum for 10 min. Fixed cells were subsequently incubated with primary antibodies against SARC (AB9465; Abcam) and Cx43 (C6219; Sigma Aldrich), followed by nuclear staining with DAPI (Sigma Aldrich). Immunofluorescence images were captured using a ZEISS inverted fluorescence microscope (Germany).

#### *In vitro* electrical cell propagation by Calcium (Ca^2+^) imaging

The electrical activity and intracellular calcium (Ca²⁺) signaling of CMs cultured with the prepared hydrogels were evaluated using (Ca²⁺) imaging. H9C2 CMs were immersed with 2.5 mM cell-permeant Fluo-8 AM (Abcam, UK)) at 37 °C for 15 min according to the manufacturer's protocol. After loading, 100 µL of stained cell suspension was placed into a microscope chamber, and the hydrogel sample was gently positioned above the cell layer. Pacing electrodes were placed on both ends of the hydrogel, and an electrical current was applied through the hydrogel. The culture medium was removed during stimulation to ensure current transmission occurred solely via the hydrogel and not through the surrounding medium. Electrical pacing was performed at a frequency of 1 Hz with parameters of 20 V and 1 ms pulse width. Under these conditions, the CMs exhibited distinct rod-shaped morphology with rhythmic contractility, confirming physiological responsiveness. Ca^2+^ transients were recorded under 1 Hz electric field stimulation using a C-Pace EP system. Each observation was conducted for a total of 60 sec, consisting of three sequential phases: 20 s pacing, 20 s rest, and 20 s pacing. Fluorescence signals were visualized using a Nikon ECLIPSE inverted microscope equipped with a 20× S-Fluor objective lens. Data were analyzed using ImageJ software (NIH, USA) with the Nikon ND2 Reader plugin for image sequence processing.

### *In vivo* acute myocardial infarction model

#### Animal grouping and injectable surgery model

Adult male SD rats (210-260 g, n = 30) were used for this study. All experimental procedures were approved by the Institutional Animal Care and Use Committee of Guangdong Provincial People's Hospital, PR China, in accordance with the National Institutes of Health Guide for the Care and Use of Laboratory Animals (Project Title: *Fabrication of Conductive Novel Hydrogel to enhance angiogenesis in cardiac repair after Acute myocardial Infarction*; Ethical Approval No: Ky-Z-2021-570-01).

The animals were randomly assigned into five groups (n = 6 per group): Group 1 (sham), Group 2 (AMI model), Group 3 (ICon-Hyd), Group 4 ICon-O_2_-Hyd), and Group 5 (Exo@ICon-O_2_-Hyd). The AMI model was established as previously described. Briefly, rats were anesthetized with isoflurane gas at 3-5 mL/min for induction and maintained at 2 mL/min throughout surgery. After confirming adequate anesthesia, the animals were endotracheally intubated and mechanically ventilated with room air using a small-animal ventilator (Harvard Apparatus, MA, USA). The thoracic area was shaved and disinfected with 70% ethanol before surgery. A left thoracotomy was performed at the fourth intercostal space to expose the heart, and the ribs were gently retracted to visualize the left ventricle. A reference line was drawn between the junction of the pulmonary artery and the right margin of the left atrium to identify the position of the left atrial appendage. The left anterior descending (LAD) coronary artery ligated between the left auricle and the conus arteriosus using 6-0 silk suture. Successful ligation was confirmed by immediate blanching and reduced contractibility of the anterior ventricular wall, including regional ischemia. To prevent arrhythmias following ligation, 0.05 mL of lidocaine solution was applied directly to the cardiac surface. For hydrogel treatment groups, 20 µL of the prepared exosome-loaded ICon-O_2_-hydrogel was carefully injected into the infarcted myocardial region using a microsyringe, ensuring minimal disruption of surrounding coronary vessels. The sham-operated group underwent thoracotomy and cardiac exposure without LAD ligation or hydrogel injection.

#### Electrical conductivity on myocardial tissue

The electrical signal propagation within infarcted myocardial tissue was evaluated 28 days after surgery using an electrical stimulation generator (RIGOL, USA) and a physiological signal acquisition system (AD Instruments, Australia). After euthanasia, the hearts were excised immediately and the infarcted left ventricular area was isolated by carefully removing normal tissue. The tissue specimens were trimmed into 1 cm × 1 cm sections and fixed onto foam supports to ensure stable contact during measurement. Positive and negative electrodes were positioned at opposite ends of the tissue sample, and a square-wave input voltage of 0.5 V at 1 Hz was applied. The resulting electrical responses were recorded to assess electrical signal conduction across the infarcted myocardium in each experimental group.

#### Electrocardiography analysis

Electrocardiography (ECG) monitoring was performed using the PowerLab bio-signal processing system (AD Instruments, Australia) to evaluate cardiac electrical activity at 14 and 28 days post-surgery. Three needle electrodes were subcutaneously inserted into the left and right forelimbs and the right hind limb of each rat. ECG traces were recorded at a paper speed of 50 mm s^-1^, and parameters such as the duration of the QRS complex and rhythm stability were analyzed using LabChart software (AD Instruments).

#### Echocardiography

Cardiac function and ventricular geometry were assessed 28 days after treatment using a high-resolution echocardiography system (Vevo 2100, VisualSonics, Canada). Two-dimensional guided M-mode imaging was performed from the parasternal long-axis view at the mid-papillary muscle level. Quantitative parameters, including eject fraction (EF), fractional shortening (FS), end-systolic diameter (LVSd), end-diastolic diameter (LVDd), end-systolic volume (LVSv), and end-diastolic volume (LVDv), were measured to evaluate overall cardiac performance.

#### Histopathology and Immunofluorescence observations

After 28 days of treatment, the AMI-induced SD rats were anesthetized and euthanized by cervical dislocation. The hearts were immediately excised, rinsed with PBS, and fixed in 4 % paraformaldehyde at 4 °C for 24 h. Fixed tissues were dehydrated, embedded in paraffin, and sectioned into 4 µm-thick slices for histological and immunofluorescence analyses. Masson's trichrome (MTS) staining was performed to evaluate the pathological morphology of infarcted myocardium following hydrogel treatment. The infarcted area and left ventricular wall thickness were quantitatively analyzed using ImageJ software (NIH, USA). Myocardial cell apoptosis in the infarct region was detected using the TUNEL assay. Macrophage polarization (M1 and M2 phenotypes) was visualized by immunofluorescence staining with iNOS and CD206 antibodies, respectively. Cardiac-specific markers, including Cx43, α-actin, and cardiac troponin T (cTnT), were stained to examine myocardial repair and functional restoration in hydrogel-treated groups. Wheat germ agglutinin (WGA) and α-smooth muscle actin (α-SMA) staining were used to analyze cell membrane morphology and neovascularization, respectively. All fluorescence images were captured using a confocal laser scanning microscope (LSM 880, Zeiss, Germany). Quantitative analysis of apoptosis rate, myocyte size, Cx43 expression, inflammatory cell infiltration, and microvessel density was conducted based on fluorescence intensity and positive staining area.

### Statistical analysis

All quantitative data were analyzed using GraphPad Prism version 8.0 (GraphPad Software, USA) and expressed as mean ± standard deviation (SD). Statistical comparisons between two groups were performed using Student's t-tests, while and one-way analysis of variance (ANOVA) followed by appropriate post hoc tests was used for multiple group comparisons. Differences were considered statistically significant at *p* < 0.05.

## Results and Discussion

The ionic conductive injectable hydrogel was fabricated through a two-step synthesis process, as illustrated in Figure 1. Initially, the EDOT monomer was introduced into an aqueous solution containing PBA-CMC and the ionic liquid monomer [VBIM]Br in the presence of APS to initiate oxidative polymerization and generate a conductive matrix. Subsequently, covalent cross-linking occurred between pCMC, PEG-DA, and PEDOT via a Michael addition reaction between the amine groups of CMC and the acrylate moieties of PEG-DA, while PEDOT was integrated through electrostatic attraction, π-π stacking, and physical entrapment within the polymeric network. These interactions contributed to both the mechanical integrity and electrical conductivity of the hydrogel.

**Figure 2:**
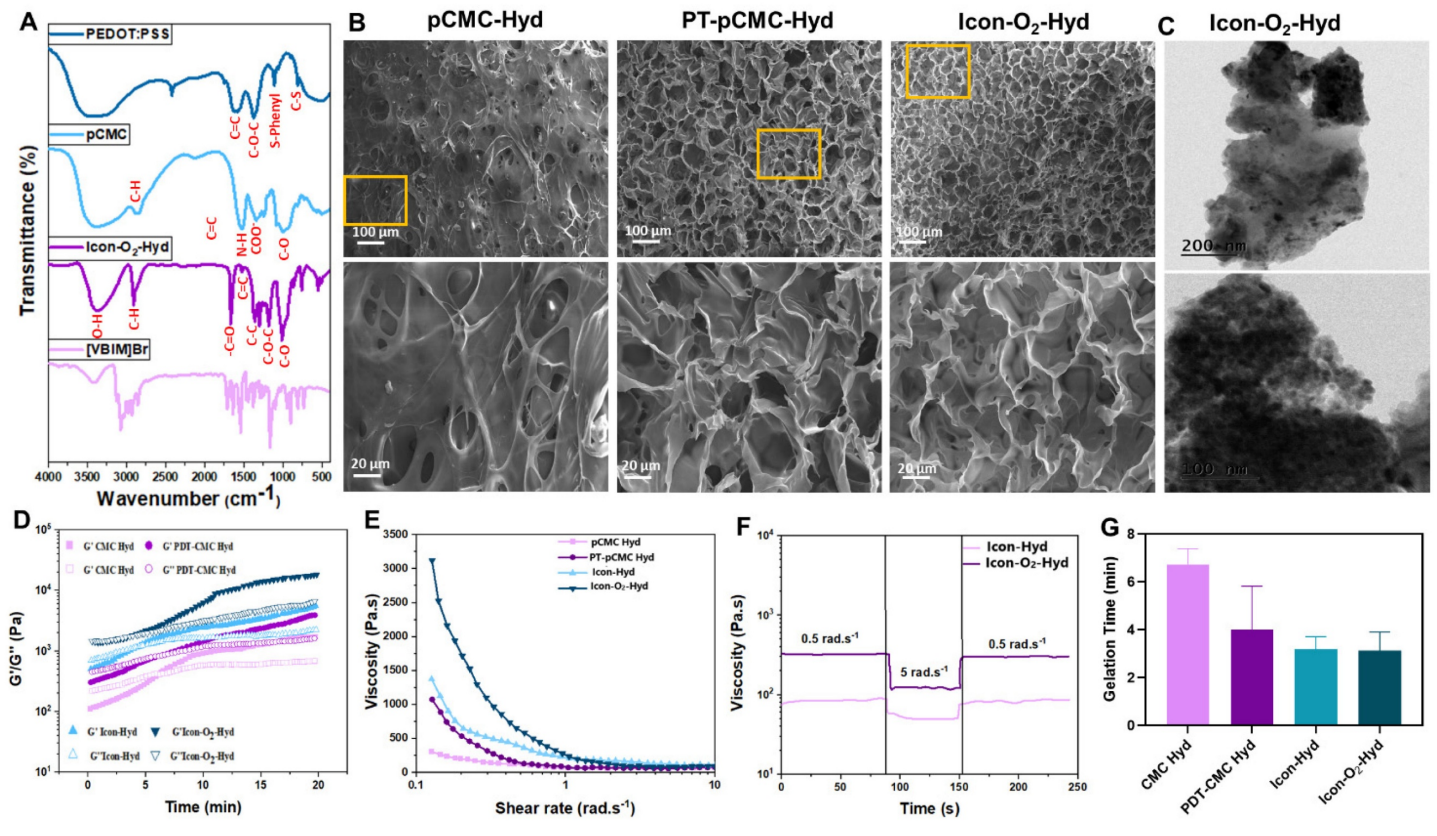
Physicochemical characterization and gelation kinetics of the conductive oxygen-generating hydrogels. (A) FTIR spectral of P-ILs, PEDOT:PSS, pCMC, and ICon-O_2_-Hydrogel. (B) Scanning electron microscopy (SEM) and (C) transmission electron microscopy (TEM) images showing the morphology and microstructure of the hydrogels. (D) Rheological time-sweep analysis illustrating gelation kinetics (G' = G”). (E) Shear rate-viscosity curves demonstrating the shear-thinning behavior of the hydrogels. (F) Step shear rate measurements assessing viscosity recovery after alternating low and high shear cycles. (G) Gelation time analysis of the prepared conductive hydrogels.

The conductive precursor solution was then mixed with PEG-DA and CaO₂ to induce gelation through physical cross-linking driven by hydrogen bonding between PBA-CMC and PEG-DA upon dissolution and cooling. The resulting hydrogel structures were analyzed using attenuated total reflectance-Fourier transform infrared (ATR-FTIR) spectroscopy to verify supramolecular interactions among the components. The ATR-FTIR spectra of PEDOT, CMC, PEG-DA, and the composite hydrogel are presented in Figure 2A. Characteristic peaks at 3450 cm⁻¹, 1630 cm⁻¹, and 1358 cm⁻¹ corresponded to the O-H stretching, C-H stretching, and C=O stretching vibrations, confirming the incorporation of PEDOT into the hydrogel network. Prominent absorption bands at 1598 cm⁻¹ and 1412 cm⁻¹ were attributed to asymmetric COO⁻ stretching and N-H bending of CMC. In addition to that, shifts in the C=O (1700-1680 cm⁻¹) and C-OH (1196-1180 cm⁻¹) bands indicated the formation of hydrogen bonds and intermolecular interactions between amine and hydroxyl groups within the hydrogel matrix, confirming successful cross-linking and structural integration of the components.

In the hydrogel preparation, EDOT (2 wt%; 200 mg) and PEG-DA (5 wt%; 500 mg) were used while maintaining a constant CMC content Higher concentrations of EDOT and PEG-DA led to the formation of solid-like structures after polymerization (Table 1). As shown in Table 1, the prepared hydrogels exhibited a more liquid and viscous appearance, indicating successful cross-linking and the establishment of a conductive hydrogel network. Notably, the conductivity of the hydrogels increased with higher PEDOT and ionic liquid concentrations. An increase in PEDOT concentration from 1wt% to 2 wt% resulted in moderate improvement in conductivity, whereas the incorporation of ionic liquids further enhanced the conductivity by approximately twofold (Table 1). The enhancement in conductivity was attributed to the intrinsic ionic conductivity imparted by the added ionic liquids within the hydrogel dispersions.

To examine the microstructural arrangements and cross-sectional morphology of the prepared supramolecular hydrogel, SEM micrographs of the lyophilized hydrogel scaffold was performed, as shown in Figure 2B. The PBA-CMC exhibited a sponge-like morphology with thicker pore walls and a reduced porous structure, displaying pore sizes of less than 10 µm. The phenylboronic acid groups formed dynamic covalent bonds with diols in the CMC polymer chains, resulting in a reversible cross-linked network that modified the internal microstructure. The incorporation of PEDOT into pCMC produced a well-interconnected porous structure, with pore walls filled by PEDOT, creating continuous conductive pathways throughout the hydrogel scaffold. The appearance of granular clusters and nodular structures indicated a denser morphology arising from PEDOT filling and the increased cross-linking density. The polymeric chains of PEDOT, carrying partial positive charges, likely interacted ionically with the negatively charged CMC, while the -OH and -NH_2_ groups of pCMC formed hydrogen bonds with oxygen atoms in the ethylenedioxy rings of PEDOT. The SEM images of hICon-O_2_-Hydrogels exhibited a uniformly arranged porous surface with an interconnected internal structure. Consistent with previous literature, the surface porosity and pore size were strongly associated with the cross-linking density, as well as by amino and aldehyde functionalization, which play a significant role in modulating pore size. The optimized pore size and interconnected porous network of the implantable hydrogel provide a favorable microenvironment for cellular repair and proliferation in myocardial tissue applications. In the present work, the incorporation of PIL into the hydrogel resulted in a denser, interpenetrated network structure, contributing to improved elasticity and mechanical integrity. Moreover, the pore structure facilitated efficient nutrient transport for cell growth and differentiation. The distribution of CaO_2_ particles within the hydrogel network was further examined by TEM, as shown in Figure 2C. BET analysis (Table 2) revealed that increasing PEDOT concentration and the addition of P-ILs markedly reduced the pore size while enhancing internal interconnectivity. The specific surface area decreased from 24.5 m^2^/g to 15.4 m^2^/g in the absence and presence of P-ILs and CaO_2_, suggesting that the inclusion of ionic liquids and CaO_2_ contributed to the formation of a more-interconnected gel network with enhanced electrical conductivity.

As shown in Table 1, different hydrogel formulations were prepared using different concentrations of PEDOT, CMC, and PEG-DA, along with the addition of P-ILs and CaO_2_, to optimize and establish structural properties suitable for myocardial applications. Optimization of gelation time, injectability, and modulus is essential to ensure compatibility with surgical implantation requirements. Under oscillatory testing at 70 min^-1^ frequency and 10 % strain amplitude, the hydrogels were evaluated to simulate the deformation microenvironment of myocardial tissue. The gelation kinetics of the prepared hydrogels were characterized by the transition from a solution (sol) state (G' < G'') to a gel state (G'>G”), as shown in Figure 2D. The gelation window (Figure 2G), was primarily influenced by the electrostatic interactions between PBA-CMC and PEG-DA, which enabled gelation within 4-8 min. PEG-DA acted as an effective cross-linker to facilitate rapid and stable gel formation. Previous studies have demonstrated that thermo-responsive gelation can occur between PEG-DA and chitosan without the need for additional cross-linkers. In such systems, hydrophobic interactions among chitosan molecules disrupt hydrogen bonding between PEG and water, promoting a sol-gel transition with increasing temperature. In general, gel-mediated physical interactions strongly accelerate the gelation process compared to covalent cross-linking, as shown in Figure S1.

Optimization of hydrogel injectability is a key factor for myocardial treatment applications. The prepare ionic-conductive hydrogel containing optimized PEDOT and CMC concentrations exhibited excellent shear-thinning behavior, characterized by a reduction in viscosity with increasing shear rate. As shown in Figure 2E, the shear rate increased markedly from 0.1 to 10 rad. s^-1^. The incorporation of P-ILs and CaO_2_ particles, along with higher PEDOT content, enhanced viscosity in the low-shear region, attributed to strong physical interactions between PEG-DA and CMC. Nevertheless, the viscosity of all hydrogel groups decreased at 10 rad. s^-1^, indicating reversible physical interactions that maintained shear-thinning capability and structural stability. Step-shear tests (Figure 2F) were conducted to evaluate changes in viscosity before and after injection across a range of 0.5 to 5.0 rad. s^-1^. The results showed an increase in shear force and rate as the conductive hydrogel passed through the syringe needle. The observed reduction in viscosity under shear stress was due to the temporary dissociation of the polymeric cross-linked network, which subsequently recovered its original viscosity after extrusion, supported by strong interchain bonding and rapid network reassociation. As shown in Figure SI 1, the injection force of primary hydrogel formulation gradually increased during the initial phase before plateauing when extruded through an 18 G clinical syringe needle at a flow rate of 1 mL.min^-1^. The required injection force ranged from 2 to 12 N, within the limit achievable by manual operation without specialized equipment, indicating ease of clinical application and reduced procedural complexity. Continuous mechanical forces generated by cardiac contractions can potentially cause delamination between the hydrogel and myocardial tissue interfaces; therefore adequate adhesive strength is essential. Adhesion tests using porcine myocardium confirmed that the hydrogel adhered firmly to the tissue surface. The interfacial bonding was attributed to a combination of covalent and non-covalent interactions, enhancing cohesion between the hydrogel and wet tissue. The incorporation of CMC and PEDOT with PEG-DA significantly improved lap shear adhesive strength through synergistic electrostatic and hydrogen bonding interactions. The ICon-O_2_-Hyd group demonstrated superior adhesive strength of approximately 5.0 kPa, as shown in Figure SI 1B. The level of adhesion supports stable interfacial coupling with the cyclically contracting epicardium, eliminating the need for additional sutures during implantation.

The cumulative oxygen release profiles of the ICon-O_2_-Hydrogel was determined by monitoring the dissolved oxygen concentration in the culture medium. The hydrogel scaffolds were incubated under *in vitro* hypoxic conditions, with and without cells, for 24 days in the presence of catalase (1 mg·mL^-1^). As shown in Figure SI 1E, the cumulative oxygen release varied significantly between scaffolds cultured with and without cells. The hydrogel scaffold without cells exhibited a greater oxygen release rate throughout the culture period compared with the cell-seeded scaffold. Both groups displayed a more than fourfold increase in oxygen release by the later time points relative to day 1, reaching approximately 30 % and 25 % on day 24 for scaffolds without and with cells, respectively. The initial oxygen release rate of Icon-O_2_-Hyd with CF cells were approximately 5 %, which progressively increased to 20 % by day 16. This enhancement was attributed to the CaO_2_-loaded hydrogel, which markedly improved oxygen release kinetics compared to the non-loaded hydrogel. Collectively, the cumulative oxygen release data confirmed that the ICon-O_2_-Hydrogel effectively supplied oxygen to the culture medium under hypoxic conditions, and that the oxygen release kinetics could be tuned through specific scaffold formulations.

**Figure 3:**
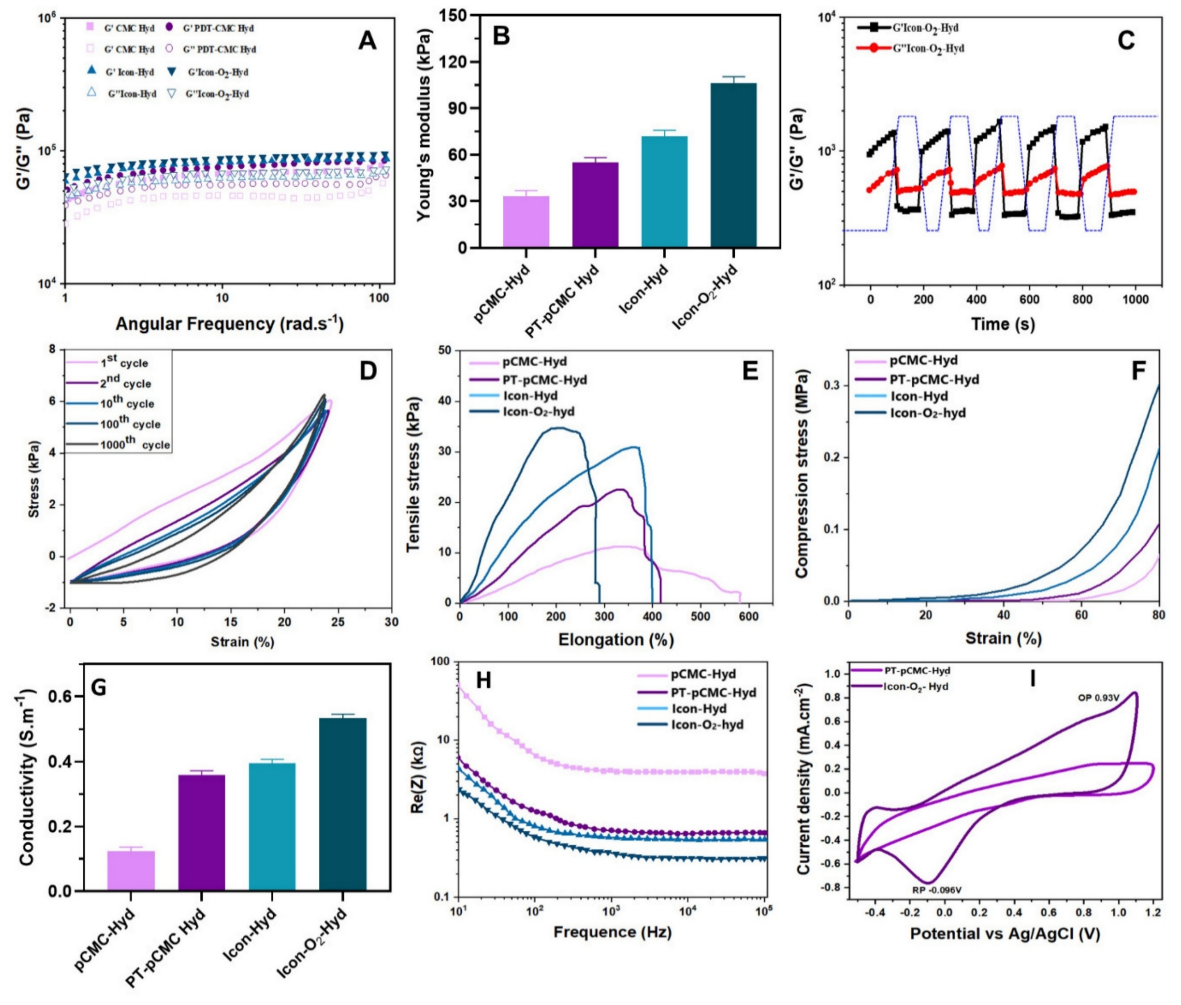
Mechanical and conductive characteristics of the developed hydrogel groups. (A) Rheological frequency-sweep analysis conducted over a frequency range of 1-100 rad.s^-1^ at normal physiological temperature (37 °C). (B) Determination of hydrogel's Young's modulus based on G' and G” values at 1 Hz (n = 3). (C) Evaluation of self-healing capability under alternating step strains of 10 % and 300 %. (D) Compression profiles of conductive hydrogel groups recorded over 1000 cycles at a compression frequency of 1 Hz. (E) Tensile and (F) compressive stress-strain curves of the prepared conductive hydrogels. (G) Measurement of electrical conductivity for each hydrogel group. (H) Electrochemical impedance spectra and corresponding frequency-dependent curves. (I) Cyclic voltammetry (CV) profiles of the conductive hydrogels showing oxidation and reduction potentials.

Viscoelastic analysis revealed that the storage modulus (G') of the primary hydrogel group exceeded the loss modulus (G”), which is consistent with the ideal viscoelastic behavior required for myocardial applications (Figure 3A). The Young's modulus of the hydrogel increased significantly with the incorporation of CMC and P-IL into the polymeric matrix, ranging from 10 to 100 kPa (Figure 3B). This modulus range closely matches the mechanical softness characteristic of biological tissues and cells (E <100 kPa). Additionally, the introduction of PEG-DA and PBA-CMC enhanced mechanical stability via strong hydrogen bonding and electrostatic interactions. The viscoelastic properties of the hydrogel were tunable through adjustments in PEG-DA and PEDOT content, as shown in Figure 3C. Mechanical evaluation indicated that the main hydrogel groups exhibited tensile strength and elastic modulus values of 10-35 kPa and 5 -12 kPa, respectively (Figure 3D). The fracture strain, compressive strength, and compressive modulus were also improved, ranging from 300-650%, 5-30 kPa, and 3-17 kPa, respectively (Figure 3E). The hydrogels displayed a high water content of 90-95 % (Table 2), suggesting outstanding compressibility and stretchability, allowing effective resistance to mechanical deformation under various microenvironmental conditions.

The prepared ICon-O_2_-Hydrogel group containing CMC and P-ILs exhibited remarkable self-healing capability, attributed to the dynamic and reversible cross-linked polymeric network. The hydrogel maintained a stable gelation state (G' > G”) under sarcomere-level strain of 10-20%, while a yield transition (G' < G”) was observed at 240%, as shown in Figure SI 2. At an oscillatory shear amplitude of approximately 300%, G′ remained consistently lower than G″, indicating temporary disruption of the network structure and decoupling of cross-links. When the strain was reduced to 10%, the hydrogel rapidly recovered to its gelation state (G' > G”), suggesting effective reformation of chemical bonds within the polymeric framework. The ICon-O_2_-Hydrogel demonstrated complete recovery of its original modulus without structural damage, confirming its rapid and robust self-healing performance. When physically separated, the hydrogel segments reattached within 2 seconds and maintained seamless integrity under subsequent stretching. This self-repairing ability is particularly advantageous in preventing microcrack propagation and maintaining mechanical continuity under cyclic stress. In myocardial applications, the maintenance of structural and mechanical equilibrium between the implanted hydrogel and native myocardium is essential in the continuously contracting diastolic-systolic environment. The dynamic borate ester bonds formed between PBA-CMC and PEG-DA facilitated spontaneous network assembly without external stimuli, thereby conferring exceptional self-healing potential. These findings indicate that ICon-O_2_-Hydrogel possesses optimal implantability, durability, and long-term mechanical stability suitable for cardiac tissue repair.

Soft biomaterials designed for cardiac implantation must establish mechanically compliant interfaces with native myocardial tissue. Accordingly, the mechanical performance of the prepared ionic-conductive hydrogels were evaluated through cyclic compression testing at 1 Hz frequency and 25 % strain, simulating the dynamic stress environment of pericardial cavity. As shown in Figure 3F, the hydrogels effectively accommodated compressive deformation, indicating controlled and stable mechanical response. The incorporation of ILs and PEDOT significantly enhanced he mechanical resilience and elasticity of the hydrogels, as evidenced by the stress-strain profiles response stabilized after an initial decay during the second compression cycle and remained steady over 1000 loading cycles, confirming the excellent anti-fatigue behavior and mechanical durability of the ICon-O_2_-Hydrogel under cyclic deformation. During cardiac diastole, implanted ICon-O_2_-Hydrogel constructs are subjected to compressive forces that can induce deformation; therefore, sufficient mechanical support is required to prevent ventricular dilation. The borate linkage-based cross-linked hydrogel network provided self-adaptive recoupling behavior, allowing synchronization with myocardial contractions while preserving stroke volume. Optimization of PBA-CMC and PEDOT concentrations, in combination with conductive ionic liquids, further improved the strength and adaptive mechanical coupling of the hydrogel matrix during myocardial relaxation. This synergy contributed to the regeneration of pulsatile function and regulation of ventricular remodelling, highlighting the tunable mechanical properties of the ICon-O₂-Hydrogel for myocardial repair applications.

The conductive polymeric hydrogel incorporating PEDOT and P-ILs effectively transmitted electrical signals through conjugated backbone structures with delocalized π-bonded electrons, demonstrating the tailored electrochemical capability of the ICon-O_2_-Hydrogel. As shown in Figure 3G, the conductivity of the ICon-O_2_-Hydrogel increased substantially from 0.4 to 0.8 S.m^-1^ compared with the hydrogel lacking ionic liquids (0.1 to 0.5 S.m^-1^), aligning with the conductivity range of electroactive tissues (0.3 to 0.7 S.m^-1^) reported in earlier studies 34-36. Additionally, the electrical resistance of the ICon-O_2_-Hydrogel decreased markedly to 450-580 Ω at higher frequencies, relative to 3350 Ω in hydrogels without P-ILs (Figure 3H). This reduction indicated that the incorporation of P-ILs within the PEDOT-based polymeric network effectively reduced charge-transfer resistance by facilitating a more continuous and homogeneous conductive phase. CV was employed to characterize the electrochemical behavior of the hydrogel for myocardial infraction therapy, providing both quantitative and qualitative insights into electrical conduction, electrochemical activity, and repetitive electrical stimulation, all essential for functional cardiac repair. Post-myocardial infarction tissues typically exhibit impaired electrical signal transmission due to cardiomyocyte loss and fibrosis; thus, the implantation of conductive hydrogels can re-establish electrical coupling between surviving CMs and restore synchronized contraction. In CV analysis, the hydrogel was positioned between working and reference electrodes, and current responses were measured under cyclic voltage sweeps. Enhanced current output reflected improved conductivity, while the CV curve shape indicated capacitive versus Faradaic behavior 34,37. The ICon-O_2_-Hydrogel exhibited a larger integral loop area and distinct redox peaks compared to other hydrogel groups, signifying superior redox activity and charge-storage capacity. The conductive PEDOT backbone within the hydrogel likely generated nanoscale electrical double layers that permitted ion penetration throughout the P-IL-rich network, facilitating efficient charge transfer through the π-π conjugated PEDOT domains (Figure 3I). The observed data confirmed that the hydrogel enabled effective charge exchange, storage, and release of physiological electrical signals. The ICon-O_2_-Hydrogel group exhibited outstanding electrochemical stability, maintaining <10 % change in charge injection capacity after multiple charge-discharge cycles (Figure SI 3A). Furthermore, piezoresistive sensitivity analysis revealed a linear current-stress relationship with a slope of 0.712 kPa^-1^ under sarcomere-level strain (Figure SI 3B-C), indicating sufficient sensitivity to accommodate rhythmic cardiac contractions. The integration of PEDOT and P-ILs established interconnected conductive pathways and participated in dynamic covalent network reconstruction, thereby achieving tissue-compatible conductivity, enhanced charge-injection capacity, high sensitivity, and reduced interfacial impedance. These characteristics collectively underscore the electroconductive potential of the ICon-O₂-Hydrogel for advanced myocardial repair in AMI treatment.

In the present report, ADSC-exos were incorporated into the hydrogel scaffolds as the principal bioactive component to promote regeneration and repair of infarcted cardiac tissue, serving as an effective alternative to direct stem cell therapy. Exosome-based therapy offers several advantages over stem cell transplantation, including low immunogenicity, high stability, ease of transport, and the absence of living cells, thereby minimizing the risk of immune rejection or tumorigenicity. Moreover, exosomes maintain their biological activity throughout the therapeutic period, making them an attractive candidate for cell-free regenerative applications. ADSCs were selected as the exosome source due to their high proliferation capacity and greater accessibility compared with mesenchymal stem cells (MSCs). Flow cytometry analysis confirmed the purity of ADSCs, showing strong expression of MSC surface markers CD29 and CD44 and the absence of CD34 (Figure SI 4A-C). Exosomes were isolated and purified from ADSCs -C). Exosomes were isolated and purified from ADSCs using standard ultracentrifugation techniques. TEM and particle size analysis revealed that the purified exosomes were spherical, with an average diameter of approximately 110 nm (Figure SI 4D-E). Fluorescence microscopy further revealed that these exosomes ere efficiently internalized by HUVECs during co-incubation, as shown in Figure SI 4F.

**Figure 4:**
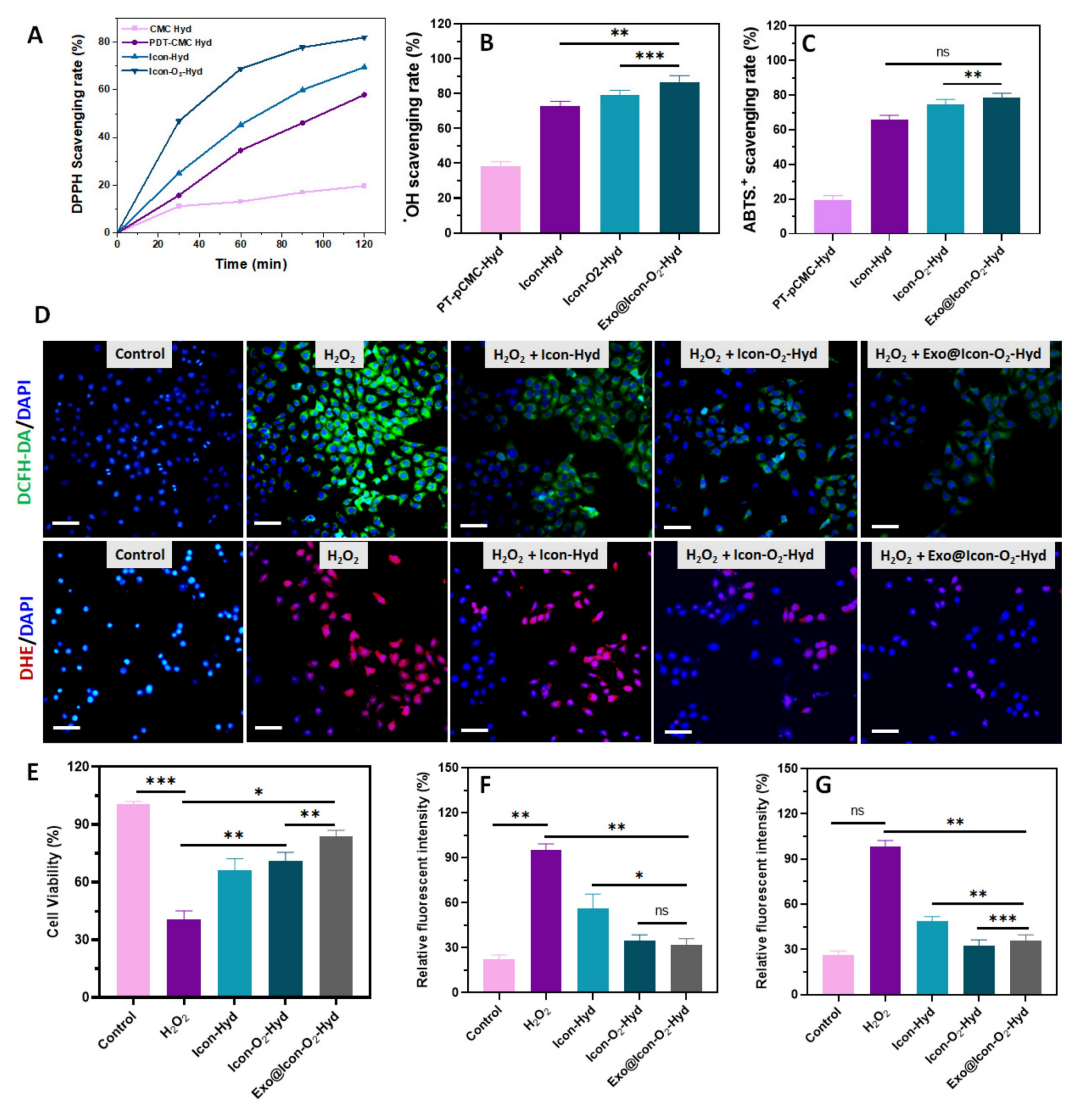
Antioxidative efficiency of the developed hydrogel samples. Scavenging efficiency of the prepared hydrogel groups against (A) DPPH^●^, (B) OH^●^, and (C) ABTS^●+^ radicals. (D) Fluorescence microscopy images of H9C2 cells treated with various hydrogels showing intracellular ROS and superoxide anion activity detected using DCFH-DA (green) and DHE (red), respectively (scale bar = 50 µm. (E) *In vitro* cell viability of H_2_O_2_-induced H9C2 cells treated with hydrogel groups assessed by CCK-8 assay. Quantitative relative fluorescence intensity measurements for (F) DCFH-DA and (G) DHE (n = 3, ns: no significant difference; *p < 0.05, **p < 0.01, ***p < 0.001).

The antioxidant capability of the prepared injectable hydrogel plays an essential role in modulating the early myocardial infarction microenvironment. In this study, a ROS-responsive PBA-CMC network was incorporated into the conductive polymeric hydrogel to improve ROS-scavenging and overall antioxidant ability (Figure 4). The antioxidant properties of the hydrogel groups were investigated using DPPH, hydroxy radicals (^●^OH), and ABTS assays, as shown in Figure 4 A-C. The presence of PBA-CMC and ADSC-exos markedly improved the the hydrogel's antioxidant capacity, with the exosomes further enhancing ROS scavenging due to their intrinsic antioxidant activity. The DPPH radical scavenging efficiencies of the ICon-O₂-Hyd and Exo@ICon-O₂-Hyd groups were 60.5% and 76.9%, respectively, relative to the control (Figure 4A). Notably, the Exo@ICon-O_2_-Hyd group demonstrated greater ROS scavenging efficiencies of 84.9% for ^●^OH and 80.7 % for ABTS^●+^ radicals (Figure 4B-C), outperforming other hydrogel formulations. To further assess antioxidant and ROS-scavenging effects on cardiac cells, *in vitro* experiments were conducted using H9C2 cardiomyocytes exposed to H₂O₂-induced oxidative stress (500 µM). As shown in Figure 4E, cell viability decreased significantly under oxidative conditions compared with the control group, confirming the cytotoxic impact of ROS. However, treatment with the hydrogel groups substantially improved cell survival, indicating that the Exo-loaded conductive hydrogels effectively protected cardiomyocytes from oxidative damage. The antioxidant performance was also visualized through DCFH-DA and DHE fluorescence staining (Figure 4D), which showed strong fluorescence signals in the H_2_O_2_-treated cells that were significantly reduced upon hydrogel treatment, confirming ROS suppression (Figure 4F-G). Overall, both qualitative and quantitative analyses demonstrated that the Exo@ICon-O_2_-Hyd and ICon-O_2_-Hyd groups effectively mitigated H₂O₂-induced oxidative stress through robust ROS-scavenging activity, thereby protecting cardiac cells in oxidative microenvironments.

**Figure 5:**
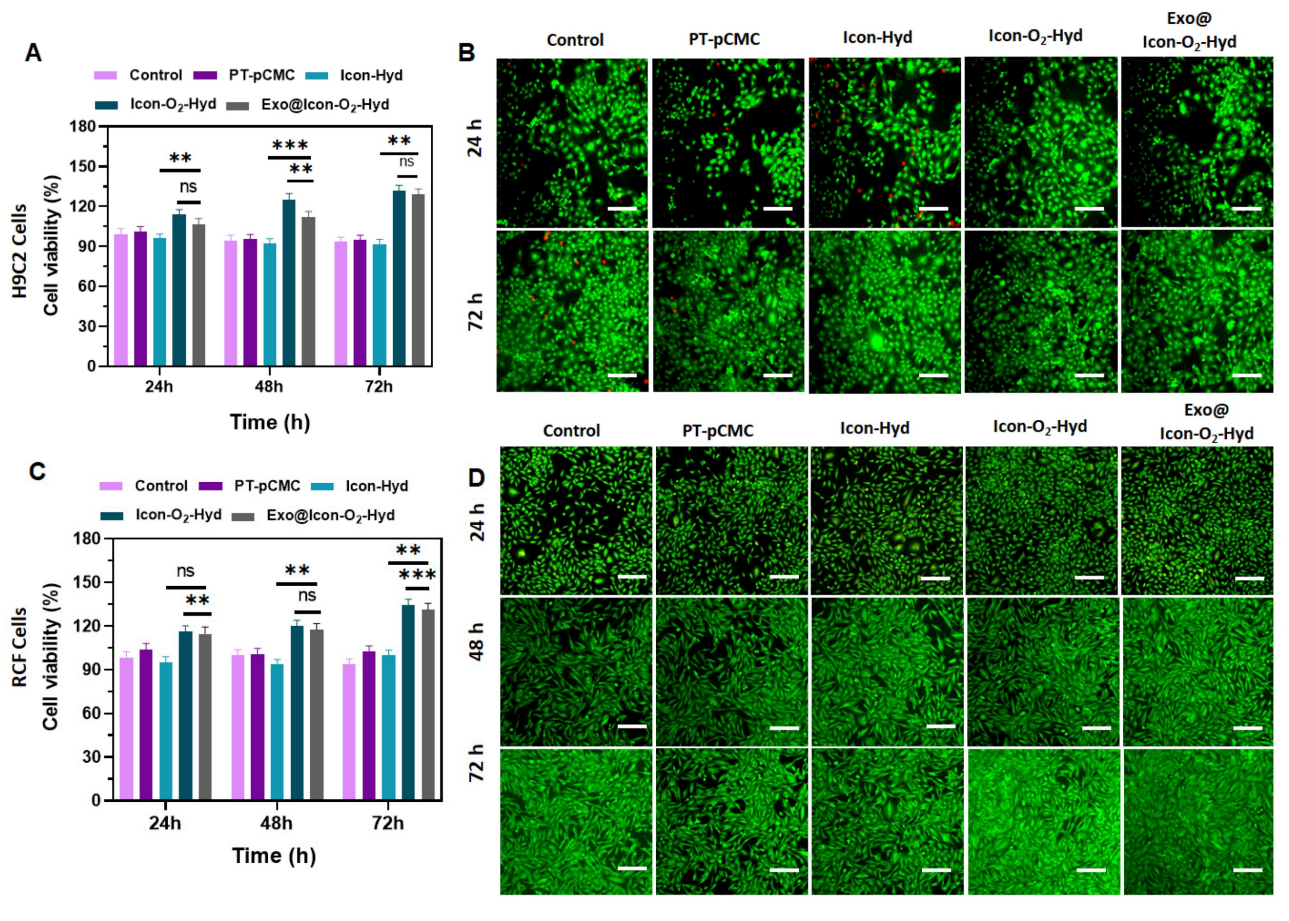
Evaluation of *in vitro* cell viability and proliferation of the prepared hydrogel groups in H9C2 cells and RCF cells. (A) Cell survival percentage of H9C2 cells treated with different hydrogel groups after 24, 48, and 72 h of incubation, assessed by MTT assay (n = 3, ns: no significant difference; *p < 0.05, **p < 0.01, ***p < 0.001). (B) Fluorescence microscopy images of H9C2 cell proliferation stained with AO (scale bar = 100 µm). (C) Cell survival percentage of RCF cells treated with hydrogel groups for 24, 48, and 72 h, determined by MTT assay. (n = 3, ns: no significant difference; *p < 0.05, **p < 0.01, ***p < 0.001). (D) Fluorescence microscopy images of RCF cell proliferation visualized using AO staining (scale bar = 100 µm).

The biocompatibility of the prepared conductive hydrogel groups was examined using H9C2 CMs and RCFs. Cell viability and proliferation, assessed through MTT and fluorescence (AO staining) assays, indicated that all hydrogel formulations supported cellular growth over extended incubation periods (24-72 h) without exhibiting cytotoxic effects. As shown in Figures 5B and 5D, the fluorescence proliferation assay revealed that the Exo@ICon-O_2_-Hydrogel group significantly improved cell proliferation at 48 and 72 h, consistent with the MTT assay results. TheExo@Icon-O_2_-Hydrogel group demonstrated superior cytocompatibility and proliferation-promoting capacity with both H9C2 and RCF cells compared to the unmodified hydrogel group. After 72 h of incubation, the cell survival rates of H9C2 and RCF cells reached approximately 120% and 126%, respectively, in the Exo@ICon-O₂-Hydrogel group, confirming the marked biological efficacy of the exosome-loaded formulation (Figures 5A & 5C). Notably, no significant difference was observed between the Exo@ICon-O_2_-Hydrogel and ICon-O_2_-Hydrogel groups, suggesting that the intrinsic biopolymeric composition and oxygen-releasing properties of the conductive hydrogel itself effectively promoted cell proliferation, even in the absence of exosomes. In addition to that, the optimized incorporation of conducting polymers and P-ILs did not adversely affect the biological activity of the hydrogel, confirming the overall biosafety and suitability of the material for cardiac tissue engineering applications.

**Figure 6:**
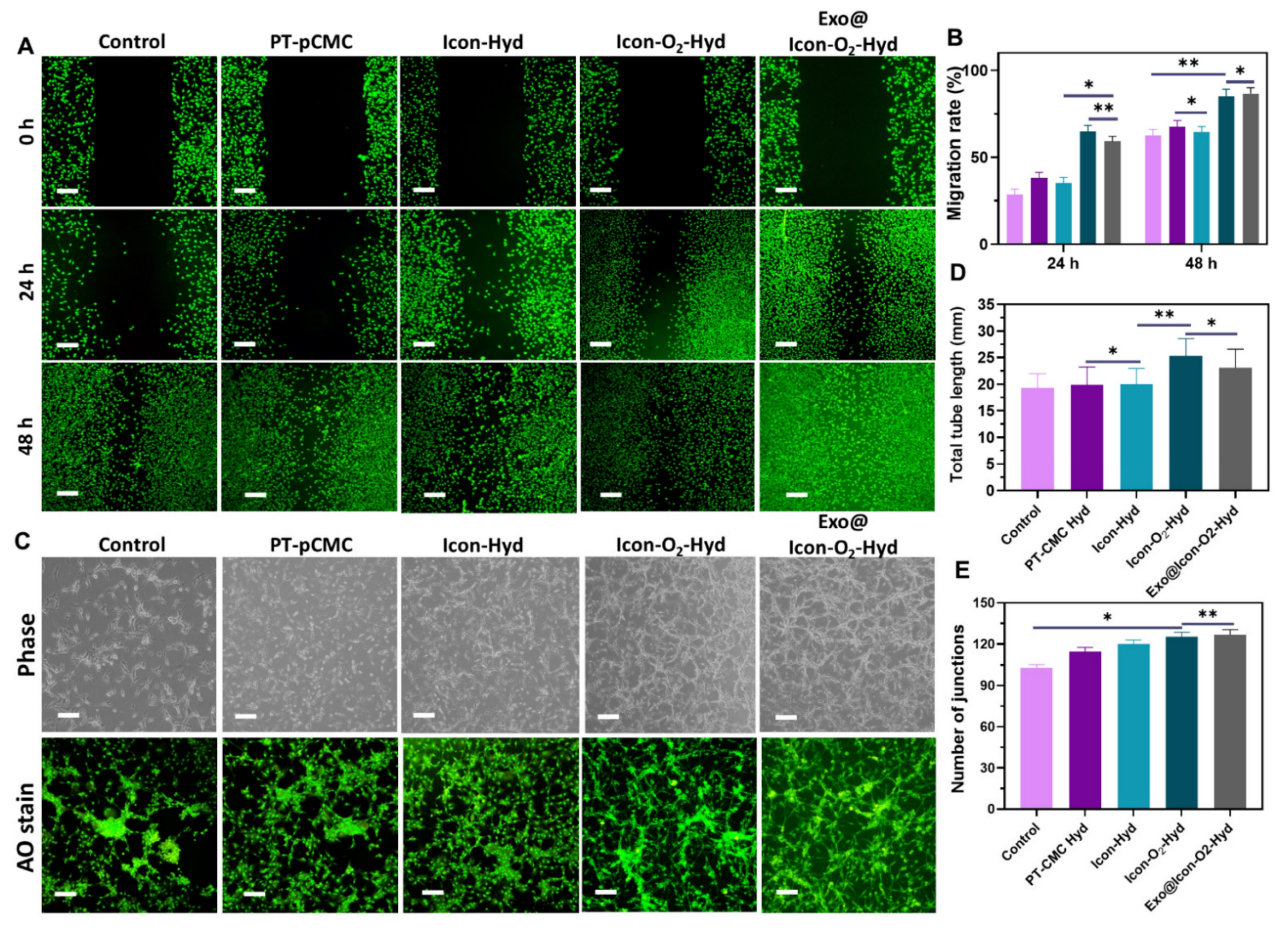
Evaluation of *in vitro* cell migration and tube formation ability of the prepared hydrogel groups. (A) Fluorescence microscopy images of AO-stained H9C2 cells showing *in vitro* cell migration at 0, 24, and 48 h following treatment with various hydrogel groups (Olympus microscope). (B) Quantitative analysis of H9C2 cell migration rate (n = 3, ns: no significant differences; *p < 0.05, **p < 0.01, ***p < 0.001). (C) Phase-contrast and fluorescence microscopy images of tube formation in HUVECs treated with different hydrogel groups for 24 h. (D, E) Quantitative measurements of total tube length and number of junctions formed by HUVECs following treatment with the hydrogel groups (n = 3, ns: no significant differences; *p < 0.05, **p < 0.01, ***p < 0.001).

**Figure 7:**
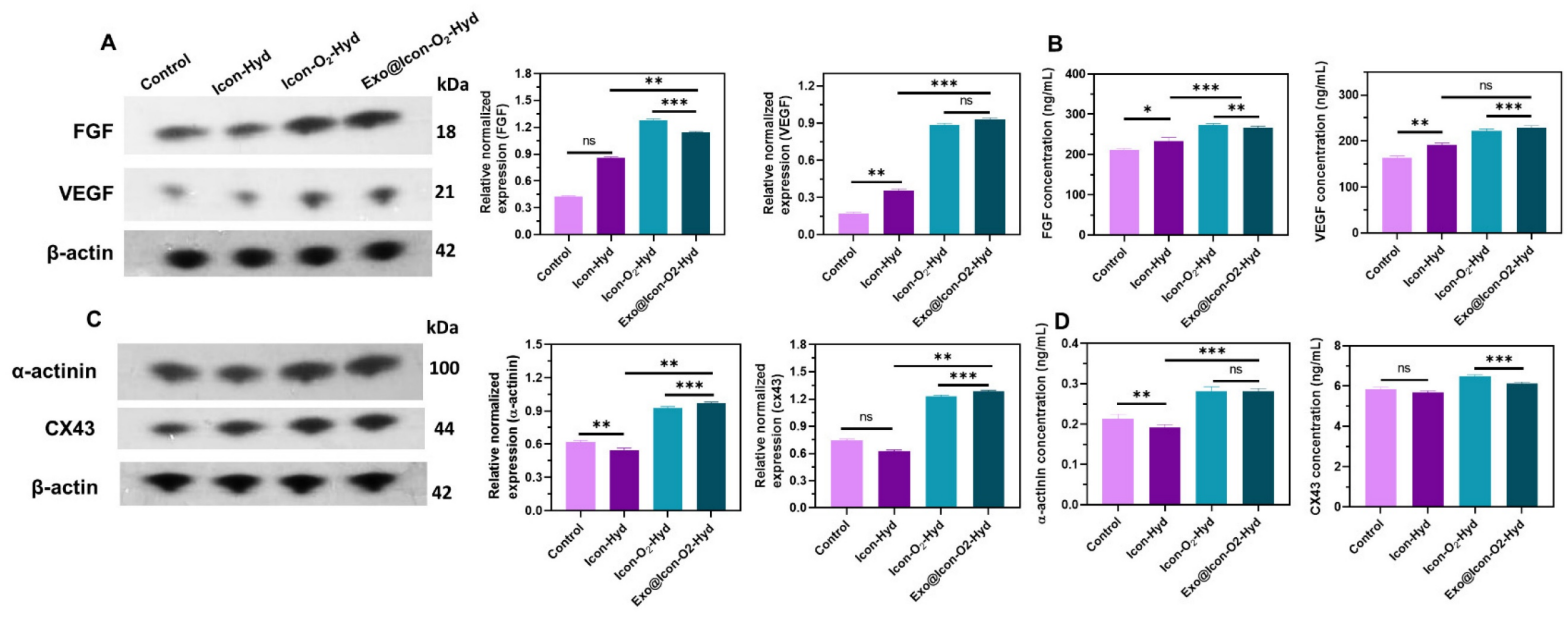
(A, B) Evaluation of FGF, BEGF, and β-actin expression levels in HUVECs treated with different hydrogel groups, analyzed by western blotting and ELISA (n = 3, ns: no significant difference; *p < 0.05, **p < 0.01, ***p < 0.001). (C, D) Evaluation of α-actinin, Cx43, and β-actin expression levels in H9C2 cells treated with different hydrogel groups, determined by the western blotting and ELISA (n = 3, ns: no significant differences; *p < 0.05, **p < 0.01, ***p < 0.001).

Disruption of oxygen supply and subsequent hypoxia often lead to extracellular matrix degradation and CM death. In this study, a CaO_2_ particle-loaded, oxygen-generating conductive hydrogel was developed to induce angiogenesis within the necrotic infarct zone by stimulating tissue regeneration from the viable border region. The angiogenic potential of the oxygen-generating conductive hydrogel was examined through a cell migration assay using cardiac RCF cells over a 48-h period. Cell migration was qualitatively observed via fluorescence microscopy with AO staining, allowing visualization of cellular movement into the scratched area. As shown in Figures 6A and 6B, the Exo@ICon-O₂-Hydrogel group exhibited significantly enhanced cell migration at both 24 and 48 hours compared with other hydrogel groups. Quantitative analysis revealed that the migration rates for the Exo@ICon-O₂-Hyd group were 74.7 % and 95.5 % at 24 and 48 h, respectively, confirming its superior migration-promoting ability relative to other hydrogels and the control. The angiogenic potential of the hydrogel extracts was further examined using an *in vitro* HUVEC tube formation assay (Figure 6C). The results demonstrated that Exo@ICon-O_2_-Hyd group effectively induced tube formation, with enhanced tube length and increased junction numbers (Figures 6D and 6E), indicating strong angiogenic stimulation. Notably, both the ICon-O₂-Hyd and Exo@ICon-O₂-Hyd groups exhibited enhanced tube formation, suggesting that the conductive O₂-generating hydrogel itself can promote angiogenesis, even in the absence of exosomes. Consistent with previous studies, exosomes released from the injectable hydrogel are known to facilitate angiogenesis, endothelial repair, and cell migration by reducing CM apoptosis and modulating cardiac fibrosis in acute AMI. Stem cell-derived exosomes are enriched with angiogenic factors that stimulate endothelial migration and vascular regeneration, resulting in extended tube length, increased branching, and improved microvascular network formation. This neovascularization restores oxygen and nutrient delivery to the infarcted region, supporting myocardial recovery 38. Western blot and ELISA analyses were conducted to elucidate the exosome-mediated angiogenic mechanism following hydrogel administration. As shown in Figures 7A and 7B, the relative expressions of vascular endothelial growth factor (VEGF) and fibroblast growth factor (FGF) were significantly upregulated in the Exo@ICon-O₂-Hyd group, consistent with previous biocompatibility findings. Both VEGF and FGF are critical regulators of angiogenesis, promoting endothelial cell proliferation, migration, repair, mitosis, vascular permeability, and the formation of new blood vessels, and effectively contribute to the positive regulation of angiogenesis, while preventing apoptosis. Additionally, cell-based experiments were conducted to determine the expression of myoblast-specific markers via western blotting and ELISA techniques. In myocardial tissue, the gap junction protein Cx43 plays a crucial role in mediating cardiac electrical impulse conduction and facilitating micronutrient exchange between CMs, as previously reported 14,39. The ELISA and western blot analyses demonstrated that treatment with exosome-encapsulated hydrogel extracts significantly enhanced the relative expression levels of Cx43 and α-actin compared with other groups (Figures 7C and 7D). The upregulation of Cx43 expression indicated improved intercellular electrical coupling and signal propagation among cardiomyocytes, while elevated α-actin levels reflected increased cytoskeletal organization and myogenic differentiation, both of which are vital for myocardial repair and synchronized contractility. Furthermore, the expression of HIF-1α was analyzed by western blotting to assess the oxygenation effects of the hydrogel treatment (Figure SI 5).

As expected, the AMI group exhibited significantly increased HIF-1α expression due to severe hypoxia, whereas hydrogel-treated groups showed markedly reduced levels, confirming the efficient oxygen-releasing capability of the CaO₂-loaded hydrogel system. The diminished HIF-1α expression suggested that oxygen supplementation from the hydrogel alleviated local hypoxia, improving the cellular microenvironment for tissue regeneration. Collectively, the outcomes of the biocompatibility assays indicated that the Exo@Icon-O_2_-Hyd groups demonstrated excellent suitability for *in vivo* application, supporting enhanced cellular proliferation and promoting effective myocardial regeneration and repair, thereby offering strong therapeutic potential for applications in AMI treatment.

**Figure 8:**
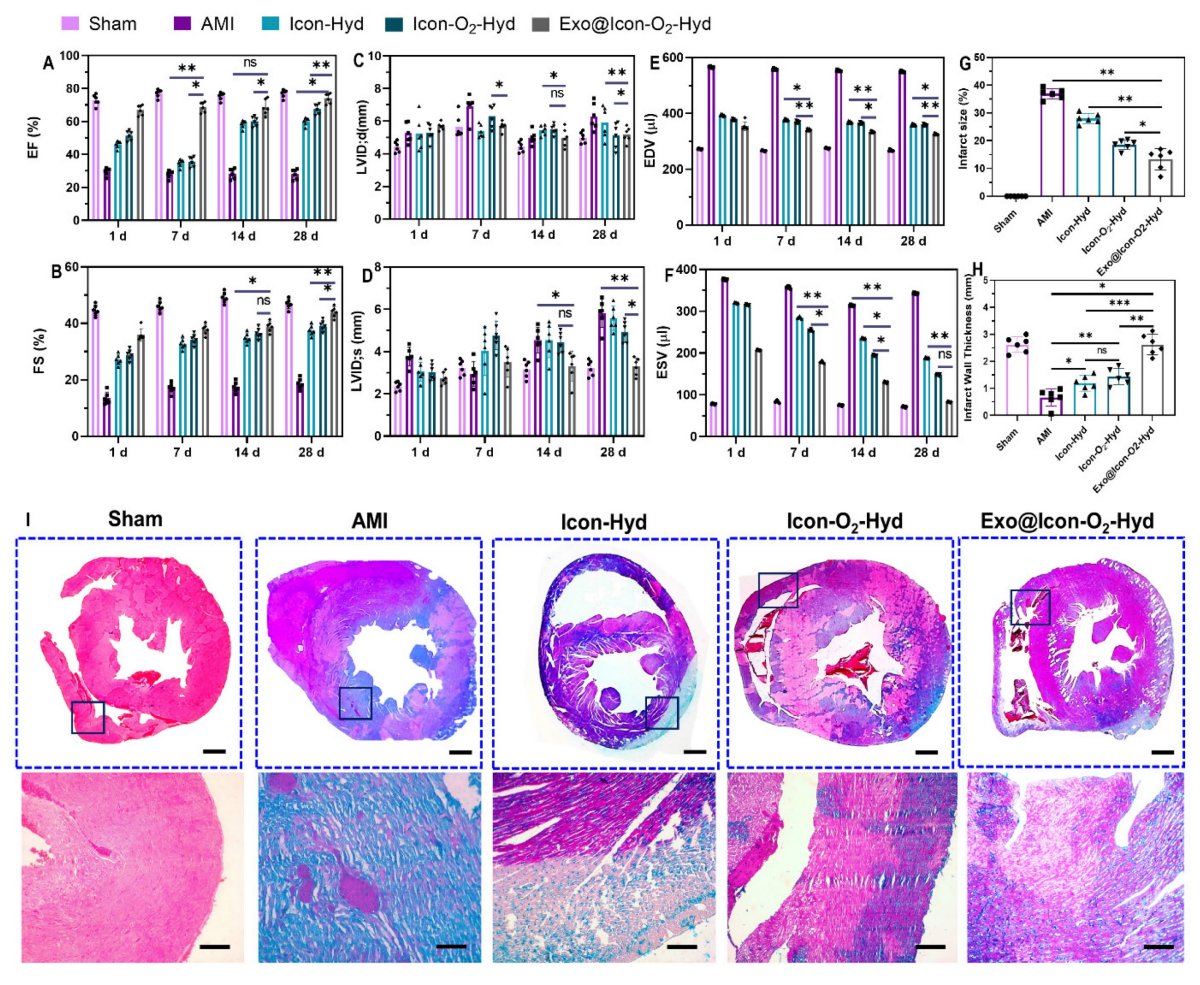
Analysis of cardiac function and histopathological evaluation of rat models treated with different hydrogel formulations. (A-F) Representative echocardiographic data depicting left ventricular cardiac function across experimental groups. (G, H) Quantitative assessment of infarct size and left ventricular wall thickness derived from Masson's trichrome (MTS) histopathological staining. Data are presented as mean ± SD (n = 6; ns: no significant difference; *p < 0.05, **p < 0.01, ***p < 0.001). (I) Representative MTS-stained cardiac sections illustrating overall heart morphology and infarcted regions in AMI and hydrogel-treated groups (scale bars: 1 mm for whole heart sections, 100 µm for magnified infarct areas).

The *in vivo* therapeutic efficacy of the exosome-loaded dual-functional conductive oxygen-generating hydrogel was evaluated using AMI-induced SD rat models. Following hydrogel administration, cardiac function was assessed by echocardiography through measurements of ejection fraction (EF) and fractional shortening (FS) at 1, 7, 14, and 28 days post-operation, as shown in Figures 8A and 8B. Both EF and FS values showed significant improvement in all hydrogel-treated groups compared with the AMI group, with the Exo@ICon-O_2_-Hydrogel group exhibiting the most pronounced recovery over time. The conductive ICon-Hydrogel group, which lacked CaO_2_ particles and exosomes, displayed moderate enhancement, whereas the incorporation of oxygen-generating particles and exosomes markedly augmented cardiac performance. These improvements are attributed to enhanced tissue oxygenation, regeneration, and restoration of electrical conductivity within the infarcted myocardium. Consistent with previous reports, injectable hydrogels can provide essential mechanical reinforcement to damaged myocardial tissue. In this study, a combined conductive and oxygen-generating therapeutic (CCOT) strategy was implemented through the fabrication of a conductive hydrogel network integrated with CaO_2_ particles for sustained oxygen release. The inclusion of ADSC-exos within the polymeric hydrogel network further supported tissue repair by activating necrotic CMs and inducing angiogenesis in the infarcted area. Comprehensive evaluation of cardiac function revealed significant differences across six key parameters—EF, FS, LVSd. LVDd, EDV and ESV—between hydrogel-treated and AMI groups (Figures 8 A-F). The hydrogel-treated rats displayed elevated EF and FS values, accompanied by reduced LVSd. LVDd, ESV and EDV measurements, suggesting improved contractility and reduced ventricular dilation. Among all formulations, the Exo@ICon-O_2_-Hyd group demonstrated the most favorable therapeutic outcomes, confirming that exosome release from the hydrogel matrix effectively enhanced angiogenesis, cardiac repair, and overall myocardial recovery in AMI treatment.

The pathological morphology, infarct size, and myocardial fibrosis of AMI-induced and hydrogel-treated cardiac tissues were evaluated using MTS histopathological staining, as shown in Figure 8(I). MTS staining revealed distinct differences in fibrotic tissue formation across the treated and untreated groups. Extensive myocardial fibrosis was evident in the untreated AMI group, indicating severe structural damage in the infarcted myocardium. In contrast, the conductive Exo@ICon-O_2_-Hydrogel group exhibited markedly reduced fibrosis and significant improvement in tissue repair compared with other hydrogel-treated groups (Figure 8A). These histological findings were consistent with the echocardiographic and cardiac functional analyses, supporting the therapeutic efficacy of the developed hydrogel. Quantitative analysis of the MTS-stained sections using Image J software (Figure 8G and 8H) confirmed that the conductive hydrogel groups significantly reduced infarct size while improving left ventricular wall thickness. The combined oxygen-generating and conductive characteristics of the hydrogel effectively contributed to fibrosis attenuation and prevention of left-ventricular dilation. The underlying mechanism is likely associated with the ability of conductive hydrogel scaffolds to form functional bridges between viable CMs in the infarcted area and surrounding healthy myocardium. In addition to that, the integration of oxygen-generating and ROS-scavenging functions facilitated improved myocardial wall thickness and reduced wall stress through activation of intracellular signaling pathways, contributing to accelerated tissue healing and regeneration. Consequently, the synergistic effect of the conductive and oxygen-releasing properties, coupled with exosome-mediated biological activity, substantially enhanced the overall therapeutic potential of the hydrogel in AMI treatment. Furthermore, the *in vivo* biocompatibility of the prepared hydrogel formulations was examined by histopathology evaluation of major organs, including the liver, spleen, kidney, and brain, using hematoxylin and eosin (H&E) staining (Figure SI 7). As shown in the histological images (Figure SI 6), no signs of congestion, necrosis, or haemorrhage were observed in any hydrogel-treated groups, whereas the AMI group exhibited pathological abnormalities indicative of systemic effects following infarction. These results confirmed that the hydrogel formulations were biocompatible and did not elicit adverse effects in major organs, supporting their safety for *in vivo* therapeutic applications.

**Figure 9:**
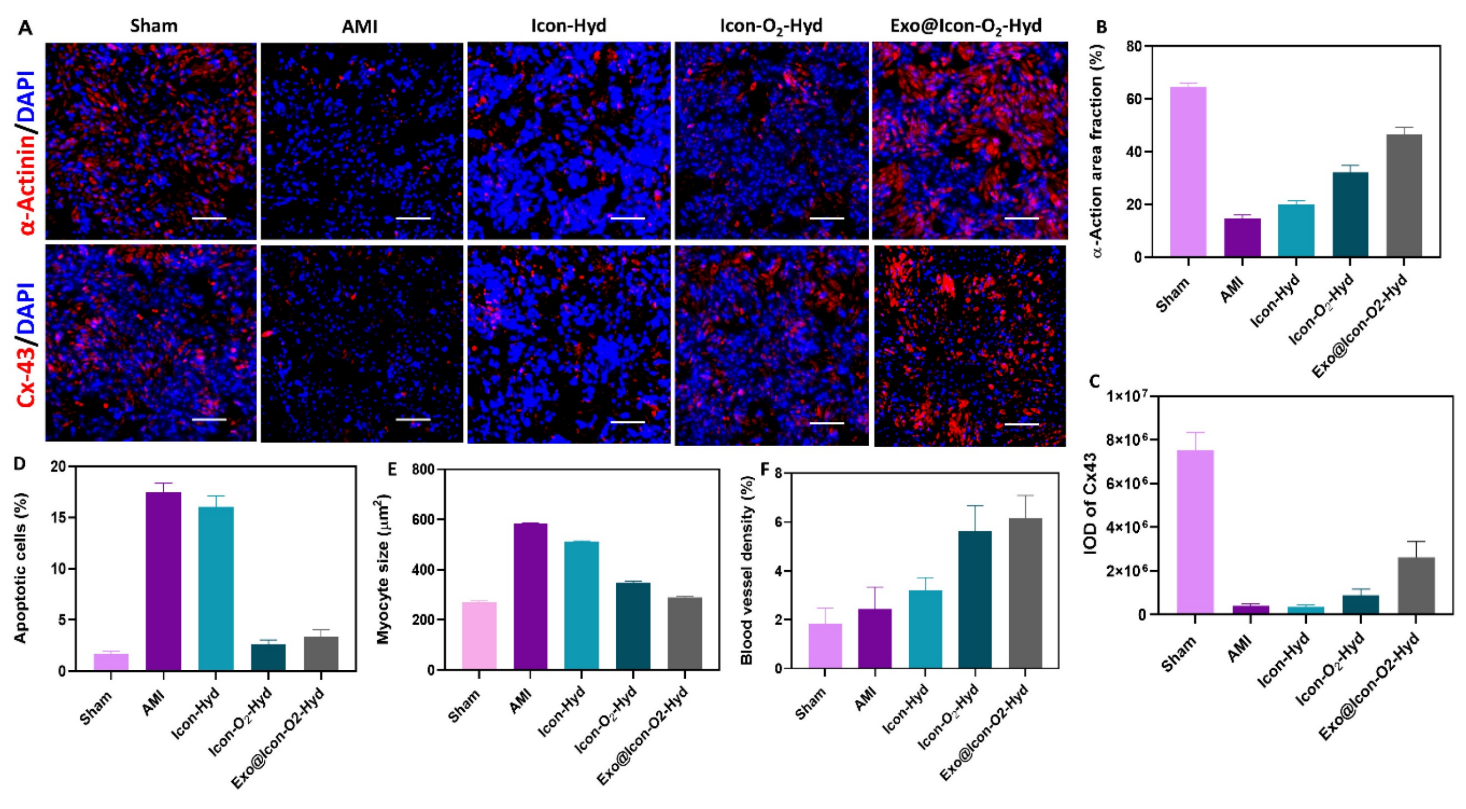
Representative immunofluorescence images of cardiac tissues treated with different hydrogel groups. (A) Expression of α-actinin and Cx43 after 28 days of treatment (scale bar = 100 µm). Quantitative observation of (B) α-actin area fraction, (C) Cx43 expression, (D) apoptotic cell percentage, (E) myocyte cross-sectional area, and (F) blood vessel density. Data are expressed as mean ± SD (n = 6; ns: no significant differences; *p < 0.05, **p < 0.01, ***p < 0.001).

The improvement of cardiac repair and structural regeneration within the infarcted myocardium was evaluated through the expressions of cardiac-specific proteins α-actinin and Cx43 using immunofluorescence microscopy (Figure 9). These two proteins play essential roles in maintaining myocardial integrity and electrical signal transmission within CMs. As shown in Figure 9A, the AMI group exhibited markedly reduced expression of α-actinin (Figure 9B) and Cx43 proteins (Figure 9C), indicating extensive myocardial damage. In contrast, both protein expressions were significantly upregulated in the conductive hydrogel-treated groups, suggesting that the hydrogel intervention effectively promoted myocardial repair and reestablished intercellular electrical connectivity. Progression of AMI typically creates a hostile microenvironment characterized by increased CM apoptosis and extracellular matrix degradation. Apoptotic cells were visualized using TUNEL staining (Figure 9D), where intense green fluorescence was observed in the AMI group, confirming extensive apoptosis. In contrast, the hydrogel-treated groups, particularly the Exo@ICon-O₂-Hyd formulation, displayed markedly reduced fluorescence intensity, indicating effective protection of CMs from apoptosis during MI therapy. Myocardial fibrosis often leads to compensatory hypertrophy of CMs; therefore, WGA staining was performed to assess myocyte size the hydrogel's capacity to preserve cardiomyocyte morphology (Figure 9E). The observed data demonstrated that the Exo@ICon-O_2_-Hyd group significantly reduced pathological hypertrophy, providing mechanical support that stabilized myocardial structure and prevented maladaptive enlargement. The myocyte size in the Exo@ICon-O₂-Hyd group closely resembled that of the sham group, confirming its ability to maintain physiological morphology while restoring mechanical and electrical function. Revascularization in the infarcted region was assessed through α-SMA expression (Figure 9F), which was substantially higher in the Exo@ICon-O_2_-Hydrogel group compared to other hydrogel formulations. This increase reflected enhanced blood vessel density driven by the recruitment, infiltration, and fusion of endogenous endothelial cells. Since revascularization and angiogenesis are critical for myocardial regeneration and CM survival, additional confirmation was obtained via CD31 immunofluorescence staining (Figure SI 6C). The positive CD31 area was significantly increased in the hydrogel-treated groups, whereas it was markedly reduced in the untreated AMI group, indicating improved microvascular network formation following hydrogel treatment. Collectively, these findings demonstrated that Exo@ICon-O2-Hydrogel effectively promoted cardiac function, reduced infarct size, and amplified angiogenesis through synergistic effects of exosome delivery and oxygen-generating conductive hydrogel network formation. Among all tested formulations, the Exo@Icon-O_2_-Hyd group showed the most prominent angiogenic activity due to the combined contribution of exosomes CaO₂-mediated oxygen release. Overall, *in vivo* data obtained from echocardiography, MTS histopathology, and immunofluorescence analyses confirmed that administration of the dual-functional conductive oxygen-generating hydrogel represents an advanced and effective therapeutic strategy for AMI repair and regeneration.

## Conclusion

This study proposed a facile combined therapeutic approach through the development of a conductive and oxygen-generating injectable hydrogel designed to improve ROS-scavenging capacity and angiogenesis for cardiac repair in AMI treatment. The ADSC-exo-loaded ICon-O2-Hydrogel exhibited prominent conductivity, mechanical adaptability, and injectability, providing a favorable microenvironment for myocardial regeneration in the infarcted area. The P-IL-enriched hydrogel, integrated with a PEDOT-based conductive network, efficiently regulated electrical conductivity to match that of native cardiac tissue, thereby restoring contractile synchronization by reestablishing disrupted electrical conduction in the infarcted myocardium. Incorporation of ROS-responsive PBA-CMC endowed the hydrogel with strong free-radical scavenging ability through dynamic phenylboronic interactions, effectively mitigating oxidative stress-induced CM injury. I*n vitro* biocompatibility analyses demonstrated that the Exo@ICon-O2-Hyd group significantly enhanced cardiac cell (H9C2 and RCF) proliferation and endothelial cell (HUVEC) angiogenic activity, facilitating tissue regeneration and vascular network formation. *In vivo* studies further confirmed that the hydrogel's outstanding mechanical-conductive coupling properties lead to reduced ventricular dilation, decreased fibrosis and wall thinning, and improved cardiac function with enhanced revascularization. Collectively, this dual-functional conductive oxygen-generating hydrogel presents a promising platform for effective cardiac repair, offering a safe and innovative therapeutic approach for clinical AMI treatment.

## Supplementary Material

Supplementary figures.

## Figures and Tables

**Table 1:** Composition and structure of prepared hydrogel dispersions

Hydrogel Label	% pCMC: PEG- DA	% PEDOT; P-ILs	Gelation	Conductivity (Ms.m^-1^)
H1	4: 2.5 %	1:1 %	Yes	85
H2	4: 2.5 %	2:1 %	Yes	230
H3	4: 5.0 %	1:2 %	Yes	460
H4	4: 5.0 %	2:2 %	Yes	758

**Table 2:** Pore size distributions of prepared hydrogel by BET analysis

Hydrogel Label	S_BET_ (m^2^/g)	V_PORE_ (cm^3^/g)
H1	24.5	0.14
H2	10.6	0.06
H3	17.4	0.12
H4	6.2	0.07
