# Supplementary material for: Fabrication of a conductive, ionic liquid functionalized, oxygen-generating hydrogel loaded with ADSC-derived exosomes to enhance angiogenesis and cardiac repair after acute myocardial infarction
Source: Theranostics. 2026 Jan 1;16(4):1997–2018. doi: 10.7150/thno.118652 (PMC12680596; doi:10.7150/thno.118652)
Supplement: Supplementary file 1 — Supplementary figures. [file thnov16p1997s1.pdf]

Supporting Information

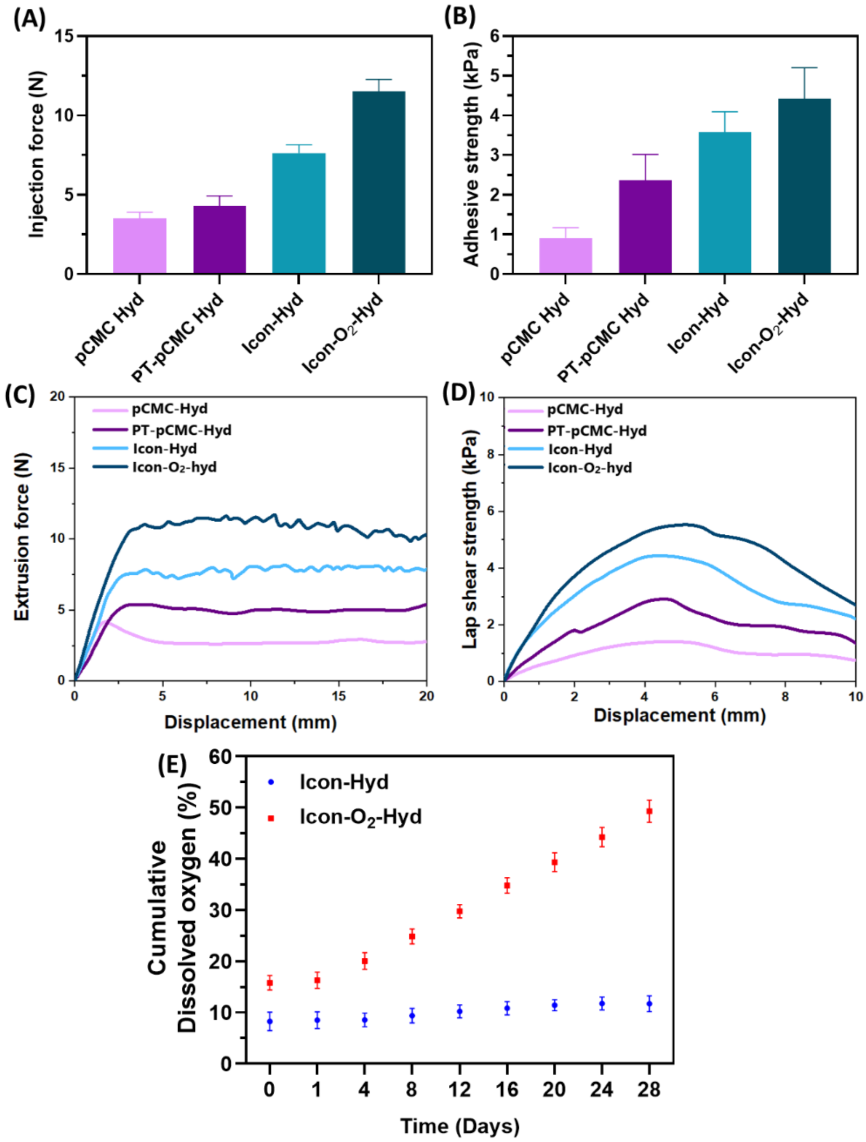

Figure SI 1. (A) The measurement of injection force by 1 mL syringe and 18G needle. (B) The analysis of adhesion strength in wet tissue with prepared hydrogel groups on porcine myocardium (n = 3). (C) The force-displacement curve on hydrogel extrusion in 18 G needle and 1 mL syringe at 1 mL.min^{-1} rate. (d) The curve of hydrogel's mechanical adhesion to porcine myocardium tissue and (E) *in-vitro* cumulative oxygen release profiles observation.

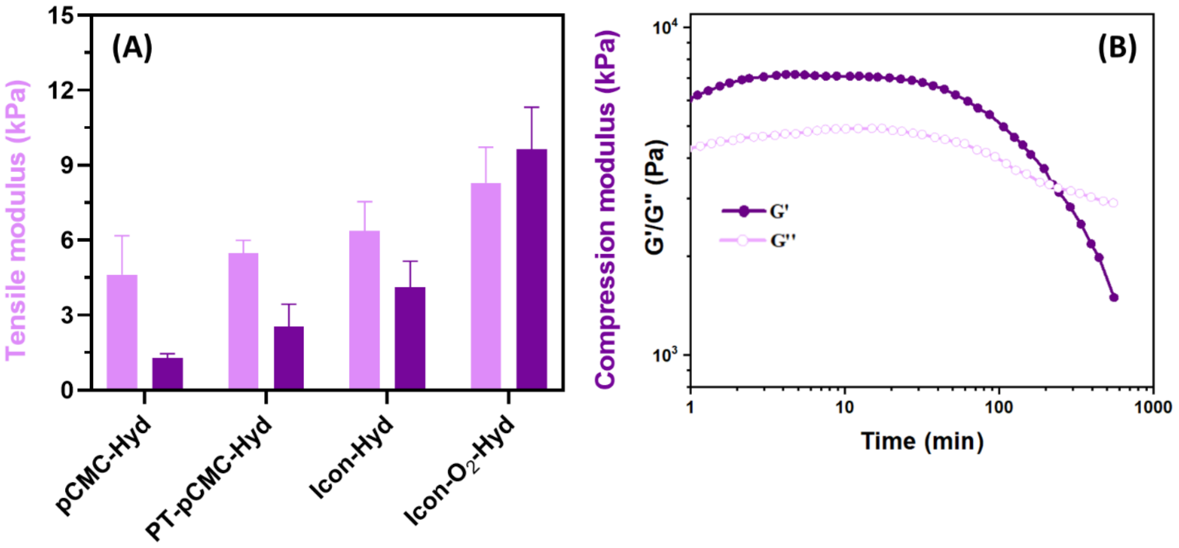

Figure SI 2. (A) Analysis of tensile modulus and compression modulus of developed hydrogels (n =3). (B) The modulus changes curves of developed Icon-O_2-Hyd group in 1-1000 % strain range.

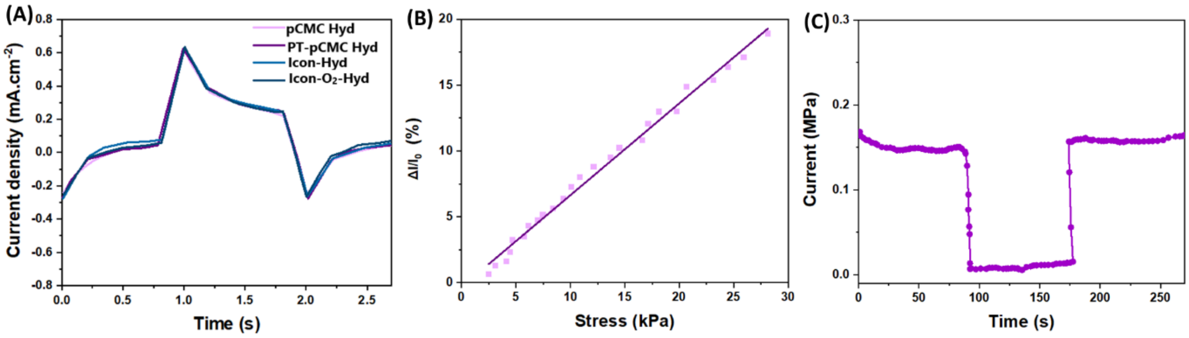

Figure SI 3. (A) Chage injection curve of prepared hydrogel groups 10 ms biphasic pulses and ± 0.5 V for 1000 cycles. (B) The Icon-O_2-Hyd group's current change curve under different compressive stress. (C) Current-time diagram for prepared Icon-O_2-Hyd hydrogel.

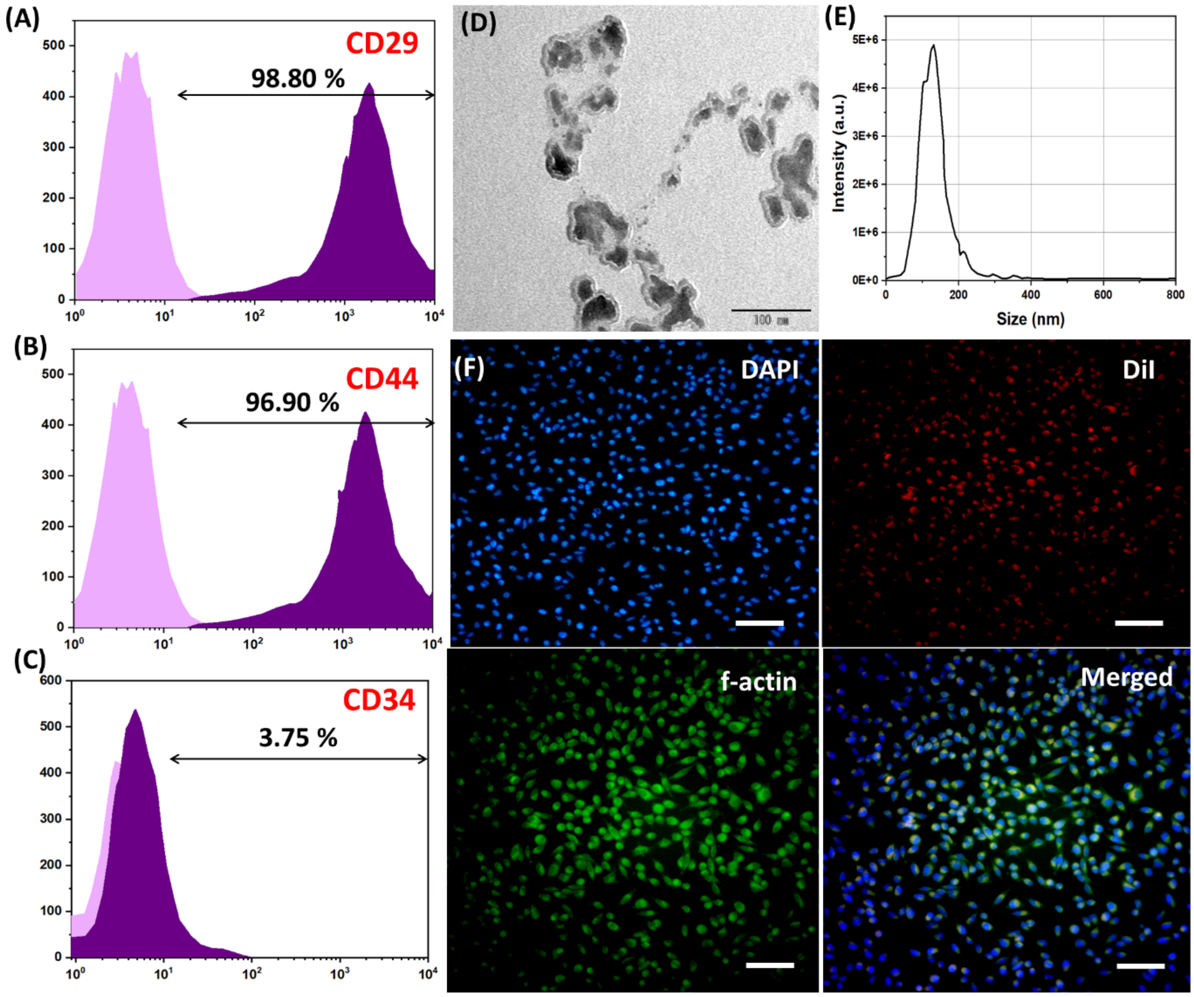

Figure SI 4. Characterizations of ADSCs-derived exosomes. (A -C) Flow cytometry investigation displayed that isolated ADSCs were positive for MSCs surface markers (CD29, CD44 & CD34). (D) TEM images and (E) particles size for ADSCs-derived exosomes. CLSM images exhibiting the endocytosis of exosomes by cells stained with DAPI, DiI and f-actin.

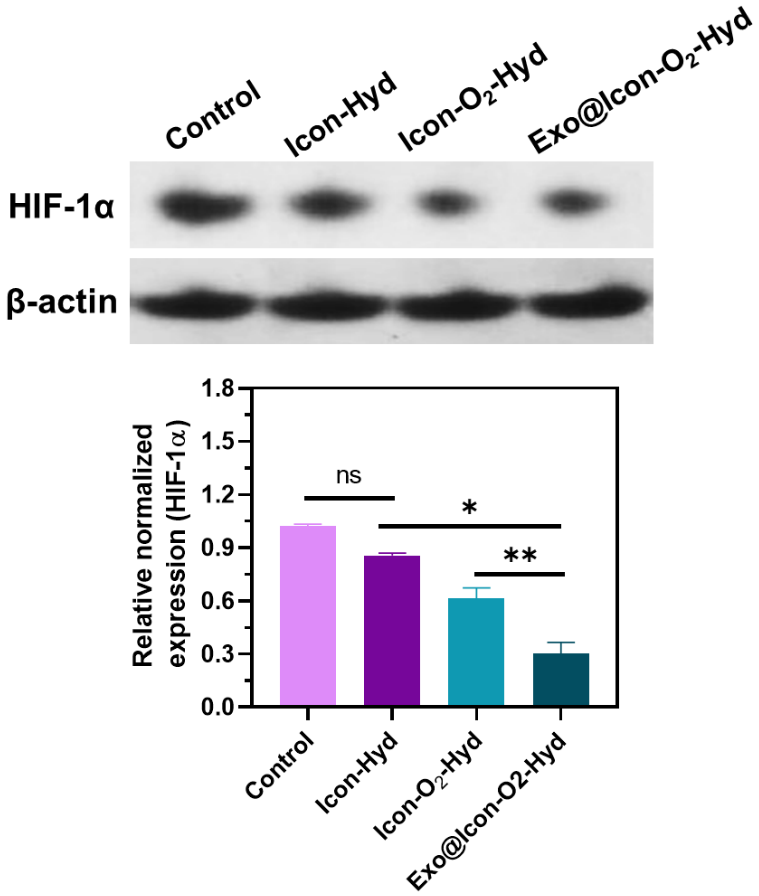

Figure SI 5. The examination of HIF-1α and β-actin expressions by the western blotting method and ELISA method on HUVECs treated with different hydrogel groups (n=3, ns: no significant differences, *p< 0.05, **p <0.01, ***p <0.001).

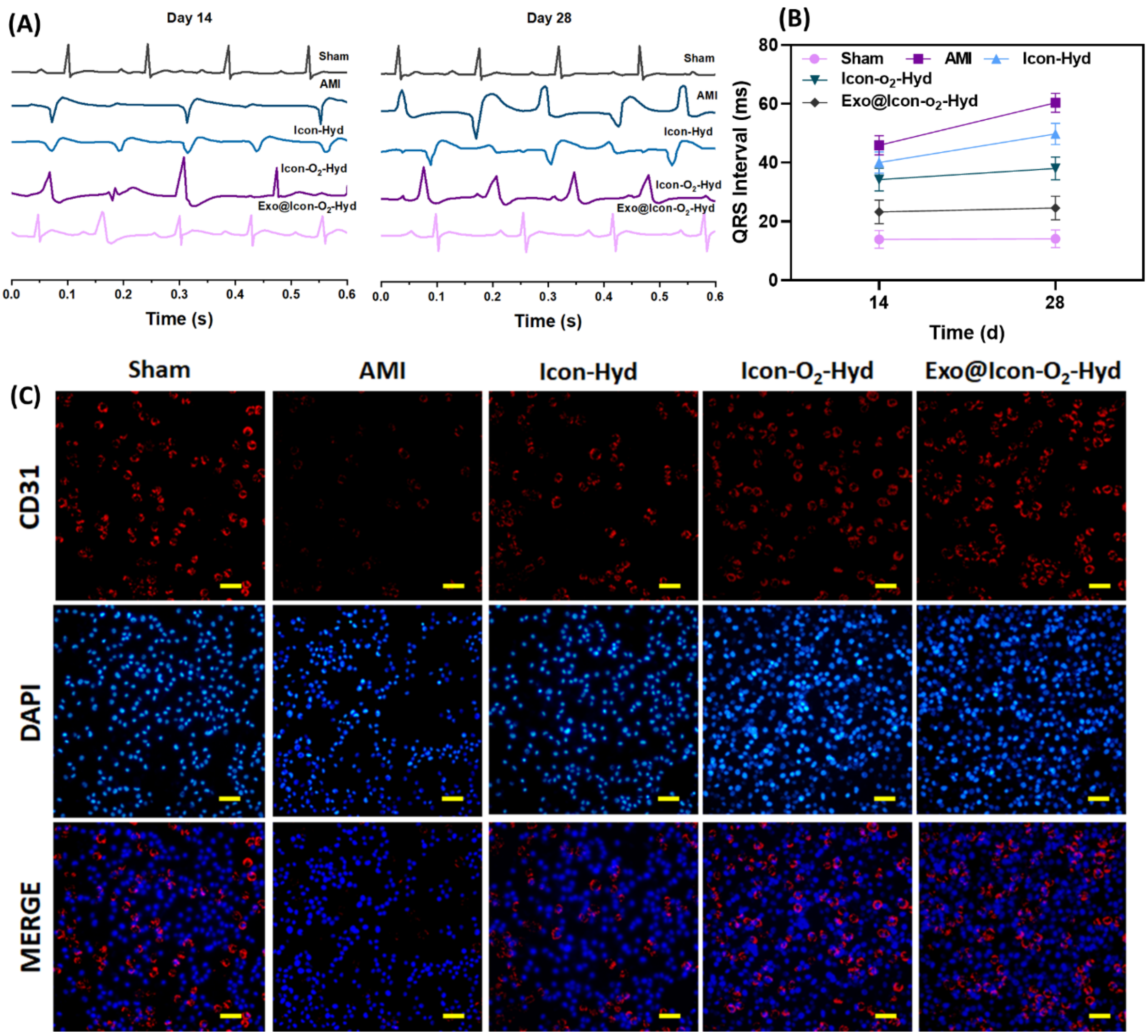

Figure SI 6. (A) The EGC analysis of AMI rat model treated with different hydrogel groups observed at 14- and 28-days post-operation and (B) quantitative measurements of QRS interval duration (n = 6) and immunofluorescence staining for CD31 (red) and DAPI (blue) of the infarcted heart tissue after hydrogel administration exhibited increased angiogenesis when compared to the AMI group (control).

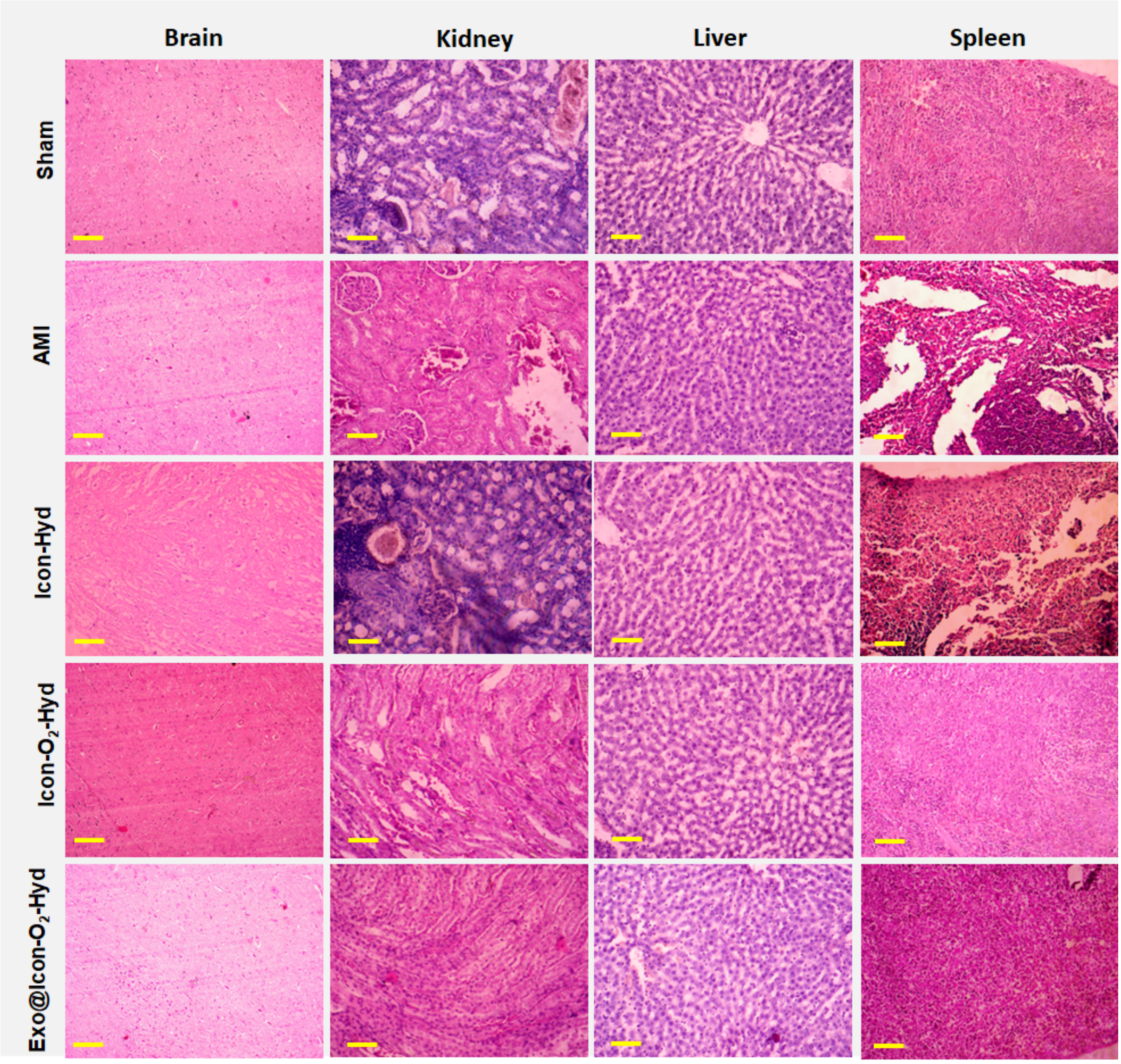

Figure SI 7. *In-vivo* acute toxicity analysis of prepared hydrogel formulation by observing major organs (brain, kidney, liver & spleen) of animal model using H&E histopathological staining (n=6), scale bar.
